# Prosaposin: A Multifaceted Protein Orchestrating Biological Processes and Diseases

**DOI:** 10.3390/cells14151131

**Published:** 2025-07-22

**Authors:** Xin Li, Liang Guo

**Affiliations:** 1 Key Laboratory of Exercise and Health Sciences of the Ministry of Education, Shanghai University of Sport, Shanghai 200438, China; lixin_012@163.com; 2 School of Exercise and Health and Collaborative Innovation Center for Sports and Public Health, Shanghai University of Sport, Shanghai 200438, China; 3 Shanghai Key Lab of Human Performance, Shanghai University of Sport, Shanghai 200438, China; 4 Shanghai Frontiers Science Research Base of Exercise and Metabolic Health, Shanghai University of Sport, Shanghai 200438, China

**Keywords:** prosaposin, saposins, Gaucher disease, Parkinson’s disease, Alzheimer’s disease, atherosclerosis, cancer

## Abstract

Prosaposin (PSAP), a multifunctional protein, plays a central role in various biological processes and diseases. It is the precursor of lysosomal activating protein, which is important for lipid metabolism and glucose metabolism. PSAP is implicated in cell signaling, neuroprotection, immunomodulation, and tumorigenesis. In neurological disorders, PSAP acts as a neurotrophic factor influencing nerve cell survival and synapse growth, and its dysfunction is associated with a variety of diseases. It modulates immune responses and macrophage functions, affecting inflammation and immune cell activities. The role of PSAP in cancers is complex, because it promotes or inhibits tumor growth depending on the context and it serves as a potential biomarker for various malignancies. This review examines current research on the functional and pathological roles of PSAP, emphasizing the importance of PSAP in Gaucher disease, neurodegenerative diseases, cardiovascular diseases, and cancer. In order to develop targeted therapies for various diseases, it is essential to understand the mechanisms of action of PSAP in different biological processes.

## 1. Introduction

Prosaposin (PSAP), also known as sulfated glycoprotein-1 (SGP-1), is the precursor protein for four lysosome-activating proteins of Saposin A, B, C, and D (Sap A–D), which promote hydrolysis of sphingolipids by lysosomal hydrolases [1]. These active peptides activate a variety of hydrolases in the lysosome and participate in lipid and glycoprotein metabolic processes. In addition to its role in lysosomes, PSAP is implicated in a variety of biological processes including cell signaling [2], neuroprotection [3], immunomodulation [4], and tumorigenesis [5].

PSAP is a multifunctional protein involved in the homeostatic regulation of many physiological processes. In the nervous system, PSAP serves as an important neurotrophic factor that promotes the survival of nerve cells, the growth of nerve synapses, and the process of differentiation [6]. Metabolically, PSAP coordinates adipose tissue energy balance and mitochondrial antioxidant capacity through lipid antigen presentation to iNKT cells and CoQ10 binding, ensuring systemic metabolic homeostasis [7,8]. It also supports reproductive physiology by interacting with Rhox5 via MAPK/PI3K-Akt pathways to regulate reproductive organ development and spermatogenesis [9]. In immune regulation, PSAP sustains lysosomal integrity and M2 macrophage-mediated immunomodulation, anti-inflammation, phagocytosis, and tissue repair to orchestrate immune equilibrium and cellular protection [10]. Furthermore, it orchestrates sphingolipid metabolism, cell proliferation, and anti-apoptosis effects through regulation of ERK, SK, and PI3K/Akt signaling, governing cellular fate determination [11,12]. These interconnected roles establish PSAP as a molecular hub integrating immune, metabolic, neural, and reproductive homeostasis.

The dysregulated function or abnormal expression of PSAP is important in the development of many diseases. In Gaucher disease (GD), PSAP-derived Sap C is critical for glucocerebrosidase (GCase, also known as glucose ceramidase or acid β-glucosidase, EC: 4.2.1.25) activity and stability, and its defects lead to a decrease in GCase function, which in turn triggers neurological lesions [13]. In Parkinson’s disease (PD), PSAP influences disease pathogenesis by affecting α-Syn levels and regulating GCase activity [14]. In Alzheimer’s disease (AD), PSAP can interact with PGRN to influence AD progression [15]. Additionally, researchers are starting to recognize the importance of PSAP in cardiovascular diseases like atherosclerosis, as it might influence the formation of plaques by impacting the function of macrophages [16]. In tumors and cancers, PSAP affects the development of a variety of cancers and tumors. For example, in breast cancer, PSAP may promote cancer progression by enhancing the Estrogen Receptor α (Erα)-mediated signaling axis [17]. In prostate cancer (PCa), PSAP may play a role in androgen receptor (AR)-dependent carcinogenesis of PCa [18]. In addition, PSAP can act as a biomarker in a variety of cancers, including gliomas [19], gastric cancer (GC) [20], colorectal cancer (CRC) [21], gallbladder cancer (GBC) [22], hepatocellular carcinoma (HCC) [23], pancreatic ductal carcinoma (PDAC) [24], malignant pleural mesothelioma (MPM) [25], and fibrosarcoma [26].

Due to the multiple roles of PSAP in different biological processes, a deep understanding of its specific mechanisms in different diseases is important for the development of new therapeutic strategies based on this molecule. The purpose of this review is to explore the roles of PSAP and its derived saposins in a variety of biological processes, as well as their pathological roles in different diseases, especially their roles in neurological disorders, cardiovascular disorders, and the initiation and progression of tumors. By comprehensively analyzing the current research progress, we hope to provide new perspectives for future studies and potential targets for the treatment of related diseases.

## 2. The Role of PSAP in Biological Processes

### 2.1. Prosaposin

**Figure 1:**
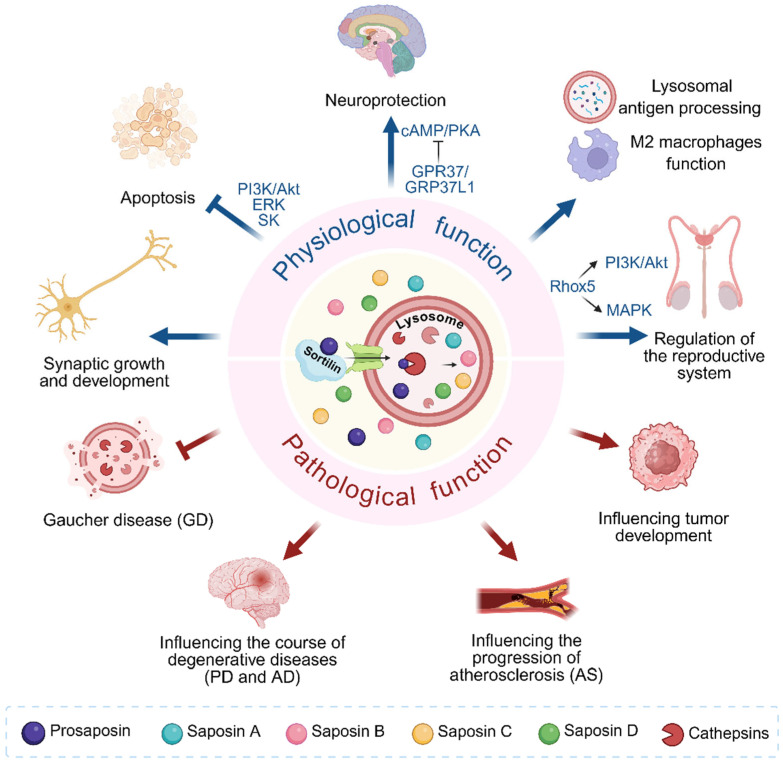
**Physiological and Pathological Functions of PSAP.** The C-terminus of PSAP interacts with Sortilin in the cell, transporting PSAP to the lysosome to activate Cathepsins, where it is hydrolyzed and processed by Cathepsins into four sphingolipid-activating proteins, known as Sap A–D. In terms of physiological functions, PSAP can promote synaptic growth and development through regulating α-synuclein (α-Syn) homeostasis. It inhibits cAMP/PKA signaling via binding to GPR37/GPR37L1 receptors, which plays a neuroprotective role in the nervous system. PSAP/Sap C binds to LRP receptors and Gα-coupled receptors to activate ERK, sphingosine kinase (SK), and PI3K/Akt signaling pathways, which inhibit TNFα expression and exert anti-apoptotic effects. PSAP may regulate M2 macrophage function and facilitate lysosomal antigen processing. PSAP also contributes to the regulation of the reproductive system: the C-terminal domain of PSAP interacts with Rhox5 via MAPK and PI3K/Akt signaling pathways, participating in prostasomal development, spermatogenesis, and regulation of fertilization capacity. In terms of pathological functions, PSAP inhibits Gaucher disease (GD). It affects the progression of degenerative diseases such as Parkinson’s disease (PD) and Alzheimer’s disease (AD), atherosclerosis (AS), and tumor development. Created with BioRender.com. (Arrows indicate promotive effects. Blunted arrows indicate inhibitory effects. Blue arrows and blue blunted arrows indicate the biological roles of PSAP. Red arrows and red blunted arrows indicate pathological roles of PSAP).

Prosaposin (PSAP) is biosynthesized, glycosylated, and extracellularly secreted, and it is hydrolyzed and processed in lysosomes by Cathepsin into four sphingolipid-activated proteins, Sap A–D [27]. Acting as activators of lysosomal enzymes is the main function of these saposins. They promote the hydrolysis of sphingolipids by hydrolytic enzymes within the lysosome and participate in the metabolic process of sphingolipids. It has been demonstrated that PSAP has different roles inside and outside the cells. Inside the cells, the C-terminus of PSAP interacts with sortilin and is transported to the lysosome, where it acts as a regulator of lysosomal enzyme function. Extracellularly, it acts as a secretory factor released into many secretory fluids such as cerebrospinal fluid, semen, milk, pancreatic fluid, and bile [3,28], and is partially located on the surface of neural protrusions as well as on the cell body [29] (Figure 1).

PSAP is an important neurotrophic factor that plays a significant role in nerve cell survival, neurite growth and differentiation [6,30]. PSAP and Sap A–D together act as ganglioside GM1 binding and transport proteins in vivo, a function that is critical for the regulation of intracellular transport and distribution of gangliosides, which in turn has an indispensable impact on processes such as neuronal differentiation, nerve growth, and synapse formation [31]. In addition, PSAP and its active peptide prosaptide (peptides containing the neurotrophic sequence of PSAP) induced an increase in ganglioside content in NS20Y neuroblastoma cells, further emphasizing the key role of PSAP in the function of the nervous system [32]. α-synuclein (α-Syn) is a protein that regulates the release of neurotransmitters in the central nervous system, and its formation of Lewy bodies is a key pathological feature of neurodegenerative diseases such as PD, leading to neuronal dysfunction and death and triggering motor symptoms and disease progression [33]. An important role in the regulation of α-Syn levels is played by PSAP and its derivative Sap C. It has been shown that overexpression of PSAP reduces α-Syn levels and helps to slow down or prevent the onset of neurodegenerative diseases, thus exerting a neuroprotective effect, whereas knockdown of PSAP leads to an increase in α-Syn levels. The above effects were independent of GCase activity [34]. Based on previous studies, G protein-coupled receptor (GPR)37 and GPR37L1 have been defined as PSAP receptors [35]. PSAP is involved in activation of its receptors GRP37 and GPR37L1 and Extracellular Signal-Regulated Kinase (ERK) phosphorylation [36]. PSAP accumulates in lysosomes of neuronal and satellite cells and co-localizes with GRP37 and GPR37L1 during development, especially in satellite cells, suggesting a role for PSAP in neuronal and satellite cell activation [37]. Here, the satellite cells are the ganglionic satellite cells that surround neurons in the dorsal root ganglion, and their main functions are to protect and provide metabolic support to neurons, and to influence neuronal development and differentiation. Astrocytes can maintain a specific state of the mitochondrial respiratory chain by metabolizing fatty acids, which ensures appropriate reactive oxygen species (ROS) signaling and maintains cognitive abilities [38]. It was shown that PSAP and Sap C were able to protect astrocytes from oxidative stress by activating GPR37 and GPR37 L1 receptors and enhance the protective effect of astrocytes on damaged neurons. Activation of these receptors in astrocytes leads to inhibition of the cAMP/PKA signaling pathway, which in astrocytes reduces oxidative stress damage and improves cellular resistance to oxidative stress, and in neuron-astrocyte co-cultures, enables astrocytes to release neuroprotective molecules to mitigate oxidative damage in neurons, thus exerting a comprehensive neuroprotective effect [39]. In summary, PSAP plays a key physiological role in neuronal survival, synaptic plasticity, and neuro-glial crosstalk by regulating ganglioside metabolism, α-Syn homeostasis, and GPR37 signaling pathways, thereby supporting the integrity and functional resilience of the nervous system.

It is well known that homeostatic regulation of adipose tissue is essential for the maintenance of energy balance and systemic metabolism [40]. *PSAP* is a key gene in the process of lipid antigen presentation, which is upregulated early in the differentiation of 3T3-L1 adipocytes and is transcriptionally regulated by CCAAT/enhancer-binding protein (C/EBP)-β and -δ. Invariant natural killer T (iNKT) cells are a distinct group of lymphocytes found in the body, known for their response to glycolipids displayed by CD1d [7]. PSAP expression in adipocytes is upregulated in synchrony with that of CD1d. And by binding to CD1d, PSAP presents lipid antigens to iNKT cells, thus playing a role in the communication between adipocytes and immune cells and regulating systemic energy metabolism and immune homeostasis [41]. Coenzyme Q (ubiquinone, CoQ) is a key lipophilic molecule in the mitochondrial electron transport chain (ETC), serving as an electron carrier in the oxidative phosphorylation (OXPHOS) process [42]. Recent studies have found that in human cells and body fluids, PSAP, a newly identified CoQ10-binding protein, plays a role in maintaining intracellular CoQ10 levels [8], especially in mitochondria [43]. This may be critical for cellular energy metabolism and antioxidant capacity. In summary, PSAP plays important roles in adipose tissue energy homeostasis, mitochondrial function, and immune-metabolic crosstalk by regulating lipid antigen presentation and CoQ10 metabolism, thereby supporting systemic energy balance and antioxidant capacity.

PSAP has also shown its importance in reproduction. Guo et al. identified the C-terminal structural domain of PSAP as a key region for the interaction with Rhox5. Through the MAPK and PI3K/Akt signaling pathways, the interaction between PSAP and Rhox5 may contribute to the development of prostasomal reproductive organs, spermatogenesis, and fertilization capacity [9].

As a key lysosomal protein, PSAP exhibits multifaceted physiological roles in immune regulation. It was shown that PSAP expression in mouse peritoneal macrophage was not significantly induced by IL-4, IL-13, or IL-10. In multiple disease models such as mouse models infected with Trypanosoma brucei, Trypanosoma congolense, Taenia crassiceps, and those bearing tumors, PSAP is upregulated in M2 (type II cytokine-associated myeloid) cells. This upregulation in vivo suggests that PSAP may have an important role in the functions of M2 cells. These functions include immunomodulation, anti-inflammatory effects, antioxidant effects, phagocytosis, and tissue repair [10]. Studies in tumor microenvironments reveal that TGF-β-induced over-glycosylation of PSAP may impair lysosomal antigen processing, and this observation underscores the intrinsic role of PSAP in maintaining lysosomal integrity and immune signaling [44]. Therefore, PSAP serves as an important regulator of immune homeostasis and cellular protection through its involvement in M2 immune cells-mediated immunologic processes and the regulation of lysosomal antigen processing.

PSAP orchestrates cell fate determination through synergistic signaling pathways. PSAP and its derivative Sap C were able to bind to U937 cells via LRP receptor and Gα-coupled receptor in a concentration-dependent manner and activate the extracellular signal-regulated kinases (ERKs) and sphingosine kinase (SK), which reduces the expression level of tumor necrosis factor alpha (TNFα). The anti-apoptotic effect of PSAP was inhibited by the PI3K inhibitor wortmannin, suggesting that the PI3K/Akt pathway may act synergistically with ERK and SK signaling to mediate cellular protection [11]. PSAP treatment also activates ERKs and SK activity in PC12 cells, increases DNA synthesis, and exerts its function in promoting cell proliferation and anti-apoptosis by increasing SK activity and sphingosine-1-phosphate (S-1-P) production, and this process can be blocked by the Mitogen-activated extracellular signal-regulated kinase (MEK) inhibitor, PD98059 [12]. Therefore, PSAP regulates cell fate by coordinating ERK, SK, and PI3K/Akt pathways, emphasizing its physiological role in sphingolipid metabolism and cellular homeostasis.

### 2.2. Structure and Function of Saposins

Saposins appear to be present in almost all tissues examined, including, brain, spleen, liver, saliva, and placenta in various species, and can be classified as “housekeeping proteins” that are essential for lysosomal hydrolysis [45]. All four saposins contain about 80 amino acids, which are structurally similar to each other, including six cysteines, a glycosylation site and conserved prolines in the same position that activate a variety of lysosomal hydrolases involved in different sphingolipid metabolisms. Despite the structural similarity of sphingolipid hydrolases, different sphingolipid hydrolases differ in their specificity and in their mode of activation [1]. Each of the four saposins will be discussed separately below.

#### 2.2.1. Saposin A

Saposin A (Sap A) is a heat-stable, 16 kDa molecular weight glycoprotein produced by PSAP proteolysis [46]. Based on X-ray crystal structures at 1.8 Å [47], 2.0 Å [48], and 1.9 Å [49] resolution, Sap A adopts a monomeric “closed” conformation in the absence of lipids, characterized by four amphipathic α-helices (α1-α4) folded into an oblate ellipsoid stabilized by three intramolecular disulfide bonds (linking α1-α4 and α2-α3), with helix α3 kinked at the conserved residue Tyr54, burying hydrophobic residues internally while exposing acidic residues on the hydrophilic surface (electrostatic potential ≈ −8.0 e). Lipids or detergents (e.g., LDAO) induce a transition to an “open” conformation through extension of helix α3 and movement of hinge regions between α1/α2 and α3/α4, enabling relative motion of “finger” structures that exposes hydrophobic residues (e.g., Trp37, Tyr30) to form dimeric discoidal complexes encapsulating a lipid bilayer (40 ordered detergent molecules) with asymmetric distribution (24 molecules upper leaflet, 16 lower) [49]. This conformational switch supports two functional models: one is the “solubilizer model” where the open conformation forms a hydrophobic cavity solubilizing diverse lipids (including phospholipids, sphingolipids, and cholesterol) [49,50] into monodisperse nanoparticles (e.g., Salipro, diameter 8.5–11.5 nm) [51], enhancing substrate (e.g., galactosylceramide) accessibility to β-galactosylceramidase [49] and stabilizing membrane proteins (increased thermal stability ΔTm = 14 °C) [52] while preserving ligand-binding capacity [53]; the other is the “liftase model” compactly encapsulating membrane proteins (adapting to 14–56 transmembrane helices) and exposing hydrophobic surfaces to facilitate lipid extraction from membranes for enzymatic catalysis [47,50,52]. Regulated by pH-dependent conformational states, Sap A remains monomeric at neutral pH (7.0) but undergoes detergent-dependent oligomerization (e.g., with C_8_E_5_) at acidic pH (4.8), forming dynamic complexes including dimers binding 23–29 palmitoyloleoylphosphatidylcholine (POPC) or tetramers binding 37–60 POPC to achieve precise lipid encapsulation control [48,54]. Sap A can increase the maximum velocity of two reactions that were sufficient to stimulate the hydrolysis of 4-methylumbelliferyl β-glucoside and glucocerebroside by, β-glucosylceramidase and the hydrolysis of galactocerebroside by β-galactosylceramidase. In addition, Sap A has no significant effect on the activities of 4-methylumbelliferyl glycoside hydrolases, GM1-β-galactosidase, or asialo-GM1-β-N-acetylhexosaminidase, and it inhibits the activity of sphingomyelinase by 30% [46]. The study indicates that Sap A facilitates the galactosylceramide hydrolysis reaction by forming a discoidal lipoprotein structure that encapsulates approximately 40 internally bound detergent molecules, solubilizing the target lipid into stable particles of around 27 kDa [49]. Another study also demonstrated that Sap A activates the degradation of glycosphingolipids in living cells, particularly facilitating the breakdown of tritium-labeled galactosylceramide [55]. Fabbro et al. [56] found by further experiments that Sap A mediates the activation effect of GCase by binding to their different sites, demonstrating that Sap A can cause conformational changes in the enzyme structure and thus increase catalysis of glucocerebroside hydrolysis.

The *PSAP* gene variant c.257T>A (p.I86N) leads to the Ile86→Asn mutation (located in the α-helix), which disrupts the structure of the hydrophobic pocket, prevents the binding of Sap A to β-galactosylceramidase and substrate localization, and triggers psychosine accumulation and a Krabbe disease-like phenotype [57]. Popovic et al. [58] revealed the key role of Sap A in the degradation of β-galactosylceramidase by introducing a specific mutation (non-glycosylated) in the *Sap A* gene in a mouse model, and found that this mutation leads to chronic globoid cell leukodystrophy (GLD, or Krabbe disease). The researchers explored the lipid extraction ability of Sap A in high bis (monoacylglycero) phosphate (BMP) and low cholesterol content membrane environments and found that Sap A was able to efficiently extract lipids from these membranes and that its glycosylation status was critical for its lipid extraction activity. Subsequent studies demonstrated that while the binding capacity of glycosylated Sap A to liposomes was diminished, its lipid extraction efficiency was augmented. This finding suggests that glycosylation plays a pivotal role in regulating the interaction of Sap A with lipid membranes and its function in lipid metabolism [59]. In addition, galactosylalkylacylglycerol (GalEAG) is a glyceride lipid containing a sugar moiety and is present as a sphingolipid in biological membranes. It was found that Sap A-deficient mice displayed significantly elevated GalEAG levels in the testis, suggesting that Sap A is critical for the in vivo degradation of GalEAG. The present findings emphasize the significance of glycosylation of Sap A for its biological functions and reveal its complex mechanisms in the regulation of sphingolipid metabolism [2].

#### 2.2.2. Saposin B

Saposin B (Sap B), also known as SAP-1, sulfatide activator protein, GM1 ganglioside activator, dispersin, and nonspecific activator [1], was the first saposin discovered by Mehl et al. [60] in 1964. The enzymes activated by Sap B are widespread, and Sap B enhances hydrolysis of galactocerebroside sulfate [61], GM1 ganglioside [62], and globotriaosylceramide [63,64]. The crystal structure of Sap B, resolved by X-ray diffraction, reveals a homodimer with each monomer adopting a V-shaped fold of four amphipathic α-helices stabilized by three intramolecular disulfide bonds (α1-α4, α2-α3) [65]. The dimer encloses a hydrophobic cavity (~900 Å^3^) lined with hydrophobic residues, enabling specific lipid binding (e.g., sulfatide) [66]. Its mechanism follows the “solubilizer model” [65]: Sap B switches between open (CC’/CD dimer) and closed (AB dimer) conformations. Conformational flexibility in the hairpin region (e.g., kink at Tyr54) and hinge loop (near residue 40) facilitates “scissor-like” motion [67,68], adjusting the cavity opening for lipid extraction. Hydrophobic interactions (M43-I46) and hydrogen bonds (Q53-Y54) stabilize the closed state [65], compressing the cavity to form soluble protein-lipid complexes that present substrates to arylsulfatase A for degradation [66,67]. It stimulates enzyme activity through interactions with substrates rather than enzymes. Li et al. [69] found that Sap B has a broad range of substrate specificity and can promote the hydrolysis of a variety of glycolipids, including glycosphingolipids such as GM1 and GM2, and non-sphingolipid glycolipids such as dihexosylglycerolipids. It exhibits natural detergent-like properties that help to disperse lipid substrates, thereby facilitating the action of glycosidases from a range of sources (microbial, plant, and animal) and increasing the efficiency of hydrolysis of glycolipids by these enzymes. Recently, it was found that PSAP with Sap B structural domain mutant resulted in reduced intracellular CoQ10 concentration due to loss of its ability to bind CoQ10, confirming the functional importance of PSAP and Sap B in intracellular CoQ10 transfer and function [70]. In terms of reproduction, Sap B may assist arylsulfatase A in the degradation of seminolipid, and a mouse model deficient in Sap B showed elevated levels of seminolipid, suggesting that it is instrumental in the metabolism of seminolipids, which may affect spermatogenesis [2].

The Lys227del mutation in the *PSAP* gene causes functional impairment of Sap B, specifically disrupting its conformational stability. Sap B serves as an essential activator protein for arylsulfatase A. When the conformational stability of Sap B is compromised, arylsulfatase A cannot effectively degrade sulfatides, even if arylsulfatase A enzyme activity is normal. This results in sulfatide accumulation in neural cells, leading to metachromatic leukodystrophy (MLD) with pathological features including central/peripheral demyelination, cerebellar ataxia, and extraneural manifestations like gallbladder polyposis [66,68]. This structural importance in Sap B function exemplifies the saposin family’s conserved mechanism for lipid metabolism regulation. Akil et al. [71] found that Sap B is critical for keeping spiral ganglion (SG) neurons healthy, and its absence causes the accumulation of sulfatide in satellite cells. This in turn triggers the degeneration of these cells and the loss of SG neurons, ultimately leading to hearing loss. In addition, it was found that a single-base mutation in Sap B leads to a defect in its glycosylation site, which in turn affects the normal catabolism of sphingolipids, resulting in tissue accumulation of cerebroside sulfate and clinical symptoms similar to metachromatic leukodystrophy [72].

#### 2.2.3. Saposin C

Saposin C (Sap C) previously termed factor P [73], glucocerebrosidase activator [74], coglucosidase [75], sphingolipid activator protein 2 [76], and A_1_ activator [77]. The crystal structure of Sap C reveals a closed monomeric conformation (2.4 Å resolution) with four amphipathic α-helices stabilized by three intramolecular disulfide bonds (α1-α4, α2-α3), where helix 3 exhibits a 64° kink at Tyr54, and hinge region flexibility (between α1/α2 and α3/α4) enhances conformational plasticity [48]. Other crystal structures show an open homodimeric conformation (domain-swapped) with dimer interfaces varying by crystal form (1721 Å^2^/monomer in orthorhombic, 1965 Å^2^ in tetragonal), where hydrophobic interactions contribute 51% to dimer stability, and hinge angles range from 112° to 116° to expose hydrophobic surfaces for membrane adaptation [48]. Its function aligns with the “liftase model”: at lysosomal acidic pH (~5.3), protonation of surface Glu residues (e.g., Glu6, Glu9) neutralizes negative charge, eliminating electrostatic repulsion with anionic membranes for pH-dependent binding (without conformational changes). Subsequent exposure of hydrophobic regions assists glucocerebrosidase in substrate degradation. Sap C activity depends on pH and lipid environment, existing as a monomer at neutral pH but forming trimers under acidic lysosomal conditions (pH 4.8) with the polyoxyethyleleglycol detergent, and this oligomerization directly facilitates membrane anchoring and lipid extraction [48,78]. In contrast to Sap B, Sap C does not interact directly with its lipid substrate but instead binds to its effector enzyme and activates it [73]. Interestingly, Morimoto et al. [46] found that Sap A and Sap C can activate the identical enzymes, β-glucosylceramidase and β-galactosylceramidase, and the fact that both enzymes are activated to a similar extent may suggest that Sap A and Sap C have homologous regions. Subsequently, they compared Sap A and Sap C in three-dimensional modeling and found significant sequence homology between them, especially in the middle regions where conserved acidic and basic residues are retained. Harzer et al. [55] added pure Sap A and pure Sap C, or a mixture of these saposins to the culture medium of living skin fibroblast cell lines from the original PSAP-deficient patients, and the galactose ceramide hydrolysis was partially restored in these cells, demonstrating that Sap A and C are activators for galactose ceramide degradation in vivo. However, the researchers concluded that it remains to be determined if Sap A and C are interchangeable with respect to their influence on galactoceramide metabolism in the whole organism.

Functional defects of Sap C lead to impaired degradation of glucosylceramide, which are implicated in the pathology of GD [48]. Meanwhile, Sap C is also a frequent target of paraproteins in GD-associated monoclonal gammopathy of undetermined significance (MGUS)/multiple myeloma [79]. Tatti et al. [80,81] found that the accumulation of autophagosomes in Sap C-deficient fibroblasts was due to delayed autolysosomal degradation, which was partially caused by reduced amounts and reduced enzymatic activity of Cathepsin B (CTSB) and Cathepsin D (CTSD), which in turn led to reduced autolysosomal degradation, impaired mTOR reactivation, and lysosomal re-formation is delayed. The study demonstrates the importance of Sap C in the regulation of autophagy and suggests a role for CTSB and CTSD in maintaining lysosomal function and autophagic homeostasis. Moreover, Sap C deficiency further impairs cellular autophagy and lysosomal homeostasis by affecting lysosome remodeling and reactivating the mTOR pathway.

#### 2.2.4. Saposin D

Saposin D (Sap D), a newly discovered heat-stable glycoprotein, is the end product of SAP and the least characterized one. Sap D exists as a homodimer with each monomer adopting a V-shaped open conformation stabilized by three conserved disulfide bonds (Cys5/Cys78, Cys8/Cys72, Cys36/Cys47); bending of hinge regions (between α1-α2 and α3-α4) increases interhelical angles from 0° to 110–120°, exposing a 12 Å × 13 Å hydrophobic cavity surrounded by 33 hydrophobic residues [82,83]. This structure enables pH-dependent conformational transitions: hydrophobic surfaces exposed under acidic pH (<5.5) preferentially bind endolysosomal membranes rich in anionic phospholipids (e.g., LBPA, PI, PS) via electrostatic interactions (mediated by Lys10/Arg17 on helix a1) [84,85]. When Sap D concentration reaches a critical value (lipid/Sap D molar ratio < 100:1), hinge movements induce membrane perturbation, converting vesicles into small anionic phospholipid-enriched particles, reflecting two synergistic functional mechanisms—the solubilizer model whereby the hydrophobic cavity directly encapsulates ceramide, solubilizing it with phospholipids into soluble nanoparticles (ceramide dissolving in the phospholipid matrix while ganglioside GM1 enriches) to act as “lipid shuttles” enhancing ceramide accessibility to acid ceramidase [83,84]; and the liftase model whereby membrane perturbation disrupts lipid bilayer integrity to expose buried lipids while the cavity extracts ceramide in situ and presents it to acid ceramidase’s active site, physically removing membrane steric barriers to directly facilitate enzymatic hydrolysis [83,85]. Protonation of surface acidic residues at acidic pH reduces repulsion with anionic membranes, specifically adapting to ceramide metabolism [83].

Chemical sequencing from its amino terminus showed a collinearity between its amino acid sequence and that of structural domain 4 of PSAP. Sap D was shown to specifically stimulate acid sphingomyelinase but had no significant effect on other hydrolases [76], and it has been associated with the activation of nerve sphingolipids and ceramides [86]. Furthermore, it was found that Sap D, a small protein generated in late endosomes/lysosomes, binds to membranes containing anionic phospholipids and induces the disruption of these membranes and the formation of smaller reorganized particles in a low pH environment. This effect is sensitive to pH, lipid/saposin ratio, and the presence of anionic phospholipids, suggesting that Sap D may play an essential role in regulating the solubilization and reorganization of anionic phospholipid membranes in late endosomes/lysosomes, and that its function may not be limited to facilitating the degradation of sphingolipids, but may also be involved in mediating phospholipid membrane transformation [85]. In summary, the structural features of Sap D, including its disulfide bond-stabilized V-shaped conformation, pH-dependent exposure of the hydrophobic cavity, and movable hinge regions, collectively determine its ability to bind to anionic membranes, its membrane perturbation effect, and its auxiliary function in lipid metabolism, fully reflecting the decisive influence of structure on function.

Despite the shared folding structures and conserved disulfide bonds among the four saposins, each possesses a unique molecular recognition code (manifested in surface charge distribution, conformational differences in key residues such as Tyr54, and hydrophobic patterns in specific regions). This recognition code dictates that each saposin specifically activates only its corresponding lysosomal hydrolase and its substrate complex, thereby precluding functional redundancy. Specifically, Sap A employs its conformational changes (e.g., extension of helix α3) and key surface residues (e.g., Trp37, Tyr30) to exclusively bind and activate β-galactosylceramidase; loss of its function (e.g., the p.I86N mutation) disrupts galactosylceramide metabolism, causing Krabbe disease, and its function cannot be substituted by Sap B, C, or D. Sap B relies on its specific hydrophobic cavity formed by the V-shaped dimer to recognize and bind lipids like sulfatide, specifically activating arylsulfatase A; its functional deficiency (e.g., Lys227 deletion) leads to sulfatide accumulation in neural cells, causing MLD, and cannot be compensated by other saposins. Sap C specifically binds and activates glucocerebrosidase through the protonation state of its surface acidic residues (e.g., Glu6, Glu9) at acidic pH; its impairment results in defective glucosylceramide degradation, causing GD, and even though Sap A can activate the same enzyme in vitro, it cannot fully replace the role of Sap C in vivo. Similarly, Sap D utilizes its exposed hydrophobic cavity in the V-shaped open conformation and key basic residues (e.g., Lys10, Arg17) to selectively bind anionic phospholipid membranes, specifically assisting acid ceramidase in degrading ceramide, and its unique function has not been demonstrated to be replaceable by other family members. Consequently, mutations in each saposin affect only their specific lipid metabolic pathways and cannot be compensated by the functions of other saposins, fully demonstrating the high specificity mediated by their recognition code mechanism.

## 3. Pathophysiological Roles of Prosaposin

### 3.1. Gaucher Disease

Lysosomes are the main degradative organelles in eukaryotic cells, enclosing a large number of acidic hydrolases capable of digesting macromolecules such as glycoproteins and lipids [87], and play multiple roles in maintaining intracellular catabolism and energy homeostasis [88]. Lysosomal dysfunction is the underlying cause of a group of metabolic disorders known as lysosomal storage diseases (LSDs). When saposins are dysfunctional, specific metabolic pathways are impaired, leading to abnormal accumulation of substrates (e.g., sphingolipids) and inducing lysosomal dysfunction-associated disorders, including Gaucher disease (GD) [89].

GD is a rare autosomal recessive disorder. It is caused by a mutation in the *GBA1* gene, which is located on the long arm of human chromosome 1 at position 1q21. This results in a greatly reduced activity of the lysosomal enzyme GCase, which hydrolyzes glucosylceramide (GluCer) into ceramides and glucose. Due to insufficient GCase activity, its substrate GluCer accumulates in macrophages, leading to symptoms such as anemia, thrombocytopenia, and bone damage [90]. GD can also lead to a wide range of related disorders, such as osteoporosis and Parkinson’s disease (PD). These may be caused by oxidative stress and inflammatory responses due to complex interconnections of downstream factors such as substrate accumulation, endoplasmic reticulum (ER) stress, unfolded protein response (UPR), dysregulation of calcium homeostasis, mitochondrial dysfunction, defects in autophagy, accumulation of α-Syn aggregates, alterations in the secretion and function of extracellular vesicles (EVs), and hyper-immunoreactivity [91]. As early as 1990, MORIMOTO et al. [92] used high performance liquid chromatography (HPLC) to quantify Sap A, C and D in the spleens of normal adults and GD patients. They found that in the spleens of normal subjects, the highest level of the four saposins was Sap D. In the spleens of patients with GD, the highest accumulation of Sap A was found (about 60-fold of normal) and the accumulation of Sap D and C was lower (17-fold and 16-fold of normal, respectively).

The important role of Sap C in regulating GCase function has been demonstrated in previous studies. Sap C mediates the interaction of GCase with lipid surfaces containing moderate amounts of anionic phospholipids by disrupting the organization of lipids in lysosomal membranes [93,94,95,96,97]. To demonstrate the importance of Sap C in GD model mice, Sun and his colleagues [13] utilized a knock-in point mutation (cysteine to proline) in exon 11 of the *PSAP* gene to establish mice selectively deficient in Sap C (Sap C^-/-^), with levels of PSAP and Sap A, B, and D proteins approaching those of the wild-type. One year later, Sap C^-/-^ mice exhibited hindlimb weakness and progressive ataxia, with a marked decrease in neuromotor activity and impaired long-term enhancement of hippocampal function. Sap C deficiency resulted in modest increases in the levels of GluCer and lactoceramide (LacCer) and their deacylated analogs, confirming that Sap C has several roles in glycosphingolipids (GSL) catabolism and prominent functions in central nervous system. Sap C deficiency resulted in decreased GCase activity and protein levels, confirming the role of Sap C as a protection factor for GCase in the lysosome. In another study, scientists generated two Sap C mutant mouse models: one with a single missense mutation (C384S) in the Sap C structural domain (Sap C^-/-^), and another harboring a compound heterozygous mutation combining C384S with a null PSAP allele (PSAP^-/C384S^). Early Sap C^-/-^ and PSAP^-/C384S^ mice all grew normally. However, they developed progressive motor and behavioral defects after 3 months of age, with the majority of the mice barely able to move at approximately 15 months of age and showing no signs of hepatosplenomegaly throughout their lives. Although no accumulation of GluCer and glucosylsphingosine was detected in the brain or liver of Sap C^-/-^ or PSAP^-/C384S^ mice, these mice exhibited progressive motor and behavioral deficits, as well as patterned loss of cerebellar Purkinje cells and extensive axonal globular degeneration, pathological features that suggest an important role of Sap C in axonal membrane homeostasis, and disruption of axonal membrane homeostasis can lead to neurodegenerative lesions in lysosomal storage disease (LSDs) [98]. Sun et al. [99] backcrossed mice with a specific point mutation (V394L/V394L) in the GCase gene, which directly impairs the catalytic ability of GCase, to mice expressing reduced levels of PSAP and saposins (4–45% of the wild-type levels, termed PS-NA). It found that 4L/PS-NA mice (the progeny resulting from crossing mice carrying the V394L mutation with PS-NA mice) showed large numbers of congested macrophages and GluCer aggregates in the liver, lungs, spleen, thymus, and brain compared to PS-NA mice. GCase activity and protein levels were reduced in the liver, spleen, and fibroblasts of 4L/PS-NA mice compared with V394L/V394L mice. This evidence suggests that reduced saposins levels increased the instability of GCases in V394L mice and that these additional reductions resulted in substantial GluCer aggregation in all tissues. The results emphasize the importance of PSAP in maintaining GCase stability and activity, and the fact that reduced levels of PSAP expression in the presence of reduced GCase activity exacerbates the accumulation of GluCer in mouse tissues to promote tissue injuries.

Sap C plays an important role not only in the activity and expression levels of GCase in mouse models of GD, but also in patients with GD. Vaccaro et al. [100] characterized the cellular properties of four patients with GD caused by mutations in the structural domain of the *Sap C* gene region. It was found that Sap C levels were significantly reduced, but fibroblasts from all four patients were able to produce PSAP and Sap A, B, and D normally. These results confirmed that GCase can exhibit its activity intracellularly only in the presence of Sap C, and that the function of Sap C could not be substituted by other saposins. A patient with GD with hepatosplenomegaly, thrombocytopenia and anemia as the main clinical manifestations was reported in China in 2017. Sanger sequencing revealed the presence of a mother-derived mutation C in exon 10 of the *PSAP* gene in the affected child. The above mutation is 1133c>G (p.Pro378Arg) in exon 10 of the *PSAP* gene, which encodes the Sap C domain of the PSAP protein. Whole genome sequencing revealed a deletion in exons 2–7 of the *PSAP* gene of the patient, which was de novo on the paternal chromosome. The pathogenic cause of GD in this patient was the double allele mutation in the *PSAP* gene [101]. In 2019, a case of a two-month-old male child with acute neuronal GD-like phenotype with a biallelic mutation in PSAP was reported for the first time in India. Exome sequencing identified a homozygous stop-gain mutation (c.G1228T, p.Glu410ter) in the *PSAP* gene, which results in the premature termination of the protein leading not only to Sap C deficiency but also to Sap D deficiency [102]. In 2022, Mohamed et al. [103] reported a rare case of Sap C deficiency caused by a de novo splice-site variant in the *PSAP* gene, resulting in a GD-like phenotype, in two Emirati families. This is the first report of 2 families in which the patients carried a *PSAP* gene of c.1005+1G>A splice-site-pure variant. Molecular genetic analysis revealed that this genetic alteration induces intron 9–10 retention and generates a premature termination codon, ultimately abolishing Sap C production. Collectively, these findings establish that both clinical observations and molecular evidence corroborate the pathogenic nature of the PSAP splice site variants.

**Figure 2:**
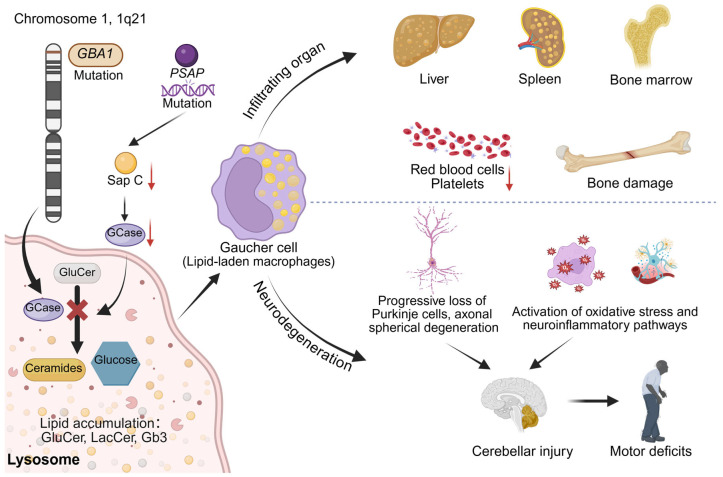
**PSAP in Gaucher disease (GD).** Gaucher disease (GD) is primarily caused by mutations in the *GBA1* gene located at chromosome 1q21. This gene encodes the lysosomal enzyme β-glucocerebrosidase (GCase), which is responsible for hydrolyzing glucosylceramide (GluCer) into glucose and ceramide. The *PSAP* gene encodes the precursor protein prosaposin, which is proteolytically cleaved to generate Saposin C (Sap C), one of the four saposins. Sap C regulates GCase function through the following mechanisms: (1) stabilizing the GCase protein structure to prevent degradation; (2) disrupting the lipid organization of lysosomal membranes to promote GCase binding to anionic phospholipid-enriched membrane surfaces, thereby enhancing its catalytic activity; and (3) directly participating in the hydrolysis of the substrate GluCer. When *PSAP* gene mutations occur (e.g., p.Pro378Arg, c.1005+1G>A splice-site variant, or biallelic deletions), Sap C production is significantly reduced or completely absent, leading to the inability of GCase to effectively bind lysosomal membranes and subsequent loss of activity. This dysfunction not only causes abnormal accumulation of GluCer but also results in widespread deposition of other sphingolipid metabolites, such as lactosylceramide (LacCer) and globotriaosylceramide (Gb3). These lipid aggregates trigger the transformation of macrophages into “Gaucher cells” (lipid-laden macrophages), which infiltrate organs such as the liver, spleen, and bone marrow, leading to anemia, thrombocytopenia, and bone damage. Additionally, Sap C deficiency disrupts axonal membrane homeostasis in the nervous system, causing progressive loss of cerebellar Purkinje cells, axonal globoid degeneration, and motor deficits (e.g., ataxia). It also activates oxidative stress and neuroinflammatory pathways, accelerating Parkinson’s disease-like neurodegenerative pathology. Created with BioRender.com.

Recently, Pavan et al. [104] reported a new case of GD with a double allele mutation in the *PSAP* gene, and they used skin fibroblasts extracted from the patient to test for GCase activity, and found that the GD patient exhibited a significant reduction in GCase activity, as well as cellular accumulation of GlcCer and/or its deacylated form GlcSph as well as accumulation of Globotriaosylceramide (Gb3) and/or its deacylated form Lyso-Gb3. From these experimental results, it is suggested that double allele mutations in the *PSAP* gene affect the production of Sap C, which in turn leads to a decrease in GCase activity, thus causing the accumulation of the associated lipids in the cell. In addition, It was found that [105] the first case of non-neurologic GD caused by Sap C deficiency. Patients exhibit have high chitotriosidase activity, elevated levels of the chemokine CCL18, increased plasma glucosylceramide concentrations, and normal GCase activity in skin fibroblasts. Molecular genetic studies of the *PSAP* gene identified a missense mutation, p.L349P, located in the Sap C structural domain and another mutation, p.M1L, located at the start codon of the PSAP. These findings demonstrate for the first time unequivocally that non-neuronal GD can be caused by Sap C deficiency. Diaz-Font et al. [106] reported a rare case of GD caused by two mutations in the *PSAP* gene, the p.C382G mutation in the Sap C region and the p.Q430X mutation in the Sap D region. The latter may lead to the generation of null alleles through nonsense-mediated mRNA decay. This is the first mutation found in the Sap D region, and mutations in the Sap D region may act by affecting mRNA stability. The above cases provide strong support that PSAP and its derived saposins are critical in maintaining GCase activity and lysosomal function, and that their defects or dysfunction may lead to a variety of biochemical abnormalities and pathological states in the nervous system (Figure 2).

In summary, PSAP and its derived Sap C play indispensable roles in maintaining GCase activity, facilitating its binding to lysosomal membranes, and mediating the degradation of GluCer. Mutations in the *PSAP* gene leading to deficiency in saposins (particularly Sap C) have been unequivocally established as key factors causing GCase dysfunction, glycosphingolipid metabolism dysregulation, and the manifestation of GD. Current research into the pathological mechanisms of PSAP/saposin-deficient GD heavily relies on specific point-mutant mouse models (e.g., Sap C^-/-^, PSAP^-/C384S^). These models successfully recapitulate some of the neurological pathologies observed in human GD patients, including progressive motor deficits, loss of cerebellar Purkinje cells, and widespread axonal globoid degeneration. However, it is important to note that these models generally fail to develop hallmark visceral manifestations of GD, such as hepatosplenomegaly and the accumulation of lipid-laden macrophages (“Gaucher cells”) within the mononuclear phagocyte system. This species-specific phenotypic discrepancy (rodent vs. human) highlights the limitations of existing models in fully mimicking the complex multi-systemic pathology of human GD, particularly the involvement of visceral organs. This gap may stem from model design (e.g., specific point mutation types, residual PSAP/saposin expression levels) and inherent species differences in metabolism and immune responses. To comprehensively dissect the precise mechanisms by which PSAP/saposin deficiency leads to systemic lipid homeostasis disruption and organ damage (especially the link between neurological and visceral phenotypes), and to lay the groundwork for developing more effective targeted interventions, future research must: develop animal models or humanized systems (e.g., mice engrafted with human hematopoietic stem cells) that better reflect the complex mutation spectrum (e.g., biallelic loss-of-function mutations) seen in human GD patients; and integrate this with in-depth lipidomic and spatial multi-omic analyses of diverse tissues (particularly brain, liver, spleen, bone marrow) from GD patients. This combined approach will elucidate how PSAP/saposins deficiency specifically disrupts lipid metabolic networks within distinct organ microenvironments to drive pathogenesis, thereby providing crucial insights to overcome current model limitations and achieve more accurate disease modeling and mechanistic understanding.

### 3.2. Neurodegenerative Disease

The saposin family (Sap A–D) serves as essential regulators of lysosomal function, facilitating substrate degradation (e.g., sphingolipids) by activating enzymes such as GCase to maintain lysosomal homeostasis. Dysfunction or aberrant expression of saposins triggers substrate accumulation (e.g., sphingolipids), impairs autophagic flux, and compromises lysosomal membrane integrity. These defects disproportionately impact metabolically vulnerable neurons. In PD, PSAP deficiency disrupts lipid homeostasis in dopaminergic neurons, exacerbating α-Syn aggregation [107], while reduced Sap C directly impairs β-glucocerebrosidase activity, promoting toxic protein accumulation [14]. In AD, saposins (e.g., Sap C) aberrantly accumulate in dystrophic neurites surrounding Aβ plaques, collaborating with loss of lysosomal hydrolases (e.g., CTSB/CTSD) to amplify pathology [15], and disrupted PSAP-progranulin interactions accelerate neurofibrillary tangle formation [108]. Thus, impaired saposins’ functional integrity is a central mechanism in neurodegenerative pathogenesis.

#### 3.2.1. Parkinson’s Disease

Parkinson’s disease (PD), characterized by progressive loss of dopaminergic neurons in the midbrain, is the most common severe movement disorder worldwide. Loss of synaptic dopamine (DA) and accumulation of protein aggregates precede the loss of dopaminergic neurons [109]. These pathologic changes lead to major clinical symptoms such as bradykinesia, postural instability, ankylosis, resting tremor, and psychiatric disorders [110,111]. A case–control association study found significantly higher allele frequencies for two variants (rs4747203 and rs885828) in the intronic Sap D structural domain region in sporadic PD patients in a combined Japanese and Taiwanese cohort. This study reveals that these variants lead to abnormal accumulation of autophagic vesicles, impaired autophagic flux, aggregation of α-Syn, and retention of PSAP in the endoplasmic reticulum, which, in turn, caused decreased motor function and degeneration of dopaminergic neurons in a mouse model, thus providing new genetic and pathological evidence for the pathogenesis of PD [112]. Using whole exome sequencing and Sanger sequencing, Chen et al. [113] analyzed the genetic etiology of 400 patients with autosomal dominant Parkinson’s disease (ADPD) and 300 patients with sporadic Parkinson’s disease (SPD). The study identified six rare and potentially pathologically significant variants located in the Sap A–D structural domain encoded by the *PSAP* gene, accounting for 0.75% of ADPD and 1.33% of SPD in the Chinese population. Using gene- and structural domain-based loading analyses, the researchers found that deleterious missense variants in Sap C were significantly associated with PD risk, while the genetic association of Sap D and PSAP with PD lacked convincing evidence. In addition, they identified an intronic variant, rs4747203, potentially associated with PSAP expression and correlated with a reduced risk of PD. To investigate the role of *PSAP* gene variants in familial Parkinson’s disease (FPD), early-onset Parkinson’s disease (EOPD) and SPD, Kuo et al. [114] summarized the relevant findings through a case–control study and meta-analysis of a Taiwanese population. The researchers sequenced exons 1–14 of the *PSAP* gene in 183 patients with FPD and 219 patients with EOPD in order to identify novel exon variants. Meanwhile, the distribution of four previously reported intronic variants (rs142614739/rs74733861, rs749823, rs4747203, and rs885828) were analyzed in 485 patients with SPD and 712 healthy controls. Two novel exonic variants in the *PSAP* gene were found to be associated with EOPD, and, in particular, intronic variant rs142614739 was associated with a significantly increased risk of EOPD.

Dysregulation of PSAP expression or function plays a key role in the pathogenesis of PD. He et al. [107] found that PSAP levels were altered in plasma, cerebrospinal fluid, and postmortem brains of patients with PD and correlated with PD-associated dyskinesias. PSAP deficiency was shown to result in reduced movement, depression/anxiety-like symptoms, and mild impairment of dopaminergic neurotransmission in a mouse model in which PSAP was specifically knocked out in dopaminergic neurons. In addition, spatial lipidomic analysis revealed an accumulation of highly unsaturated and short-chain lipids as well as a reduction in sphingolipids in the brains of PSAP-deficient mice. It was also found that overexpression of α-Syn resulted in more severe dopaminergic degeneration and higher levels of phosphorylated α-Syn, whereas PSAP overexpression by AAV or Encapsulated Cell Biodelivery (ECB) was able to protect against 6-hydroxydopamine (6-OHDA) and α-Syn toxicity in wild-type rodents. These findings suggest that PSAP plays an important role in maintaining lipid homeostasis in dopaminergic neurons, which is disrupted in PD. Furthermore, overexpression of PSAP counteracts experimental Parkinson’s disease and protects dopaminergic neurons from damage. In a recent study, researchers found that variants in the *PSAP* gene are associated with familial and sporadic PD through genetic variant screening and cell modeling studies. PSAP is involved in lysosomal function, and mutations in it may lead to aberrant accumulation of autophagic vacuoles, impaired autophagic flux, and aggregation of α-Syn. Experimental results showed that PSAP deficiency caused PD-like symptoms, including decreased motor ability and affective disorders, in a mouse model. In addition, PSAP supplementation protects dopamine neurons from toxic damage and counteracts experimental PD [115]. PSAP is a multifunctional protein that plays an indispensable role in the activation of lysosomal enzymes, with a particular focus on GCase via its derivative Sap C. Tayebi et al. [116] found that PSAP interacts with progranulin (PGRN) in lysosomes and enhances their respective transport and activation. Deficiency or mutation of PSAP results in a Sap C deficiency, which in turn reduces GCase activity and affects its normal function. In another study, researchers explored the relationship between lysosomal dysfunction and α-Syn aggregation in patients with PD, focusing on the role of PSAP and its derivative Sap C in this process. By analyzing leukocytes from 42 patients with PD and 37 healthy controls, they found that GCase and β-galactosidase activities were reduced by 20% in patients with PD compared to controls, whereas PSAP and Sap C protein levels were significantly reduced and correlated with increased α-Syn levels in patients with PD. In addition, a strong negative correlation was found between PSAP and α-Syn protein levels, suggesting that the reduction in PSAP levels promotes the accumulation of α-Syn. These results suggest that reduction of PSAP and Sap C is fundamental to promoting lysosomal dysfunction in PD and may be involved in the pathogenesis of PD by affecting GCase activity and α-Syn clearance. Therefore, PSAP and Sap C may be potential targets for PD therapy [14]. In addition, Gruschus et al. [117] found that Sap C was able to transform GCase from a multimeric state to a monomeric state, a process confirmed by analytical ultracentrifugation (AUC) and isothermal titration calorimetry (ITC). α-Syn competes with Sap C for GCase binding, but unlike Sap C, α-syn binding does not affect the multimeric state of GCase. The possible formation of stable dimeric and tetrameric forms of GCase, in which the GCase active site is partially buried, was predicted by X-ray structural analysis, suggesting that Sap C may disrupt multimeric GCase by binding to a region near the active site of GCase. These findings may have implications for the treatment of PD and GD by providing new insights into the role of Sap C in regulating GCase activity.

Kim et al. [118] found that ceramide activates CTSB, which plays a key role in mediating the decomposition of PSAP into Sap C. Sap C is an important coactivator of GCase in lysosomes. This signaling pathway is disrupted in PD-associated models, resulting in reduced GCase activity. Specifically, in Parkin knockout (KO) cells, the mature form of CTSB is reduced, PSAP processing is blocked, and Sap C levels are decreased, which in turn leads to reduced GCase activity. In addition, treatment of cells with the acidic ceramidase inhibitor carmofur increases ceramide production, which upregulates CTSB activity and promotes the processing of PSAP to Sap C, which in turn restores GCase activity. In contrast, treatment of cells with acid sphingomyelinase (ASMase) inhibitors reduced ceramide production, resulting in decreased CTSB activity, blocked PSAP processing, decreased Sap C levels, and decreased GCase activity. These results highlight a mechanistic link between ceramide and CTSB in the regulation of GCase activity and suggest that targeting lysosomal ceramide or CTSB may be an effective therapeutic strategy to activate Sap C/GCase axis in PD and related disorders. GPR37, a brain-enriched G protein-coupled receptor, plays a significant role in PD pathogenesis. Functioning as a substrate for Parkin (an E3 ubiquitin ligase), this receptor demonstrates pathological accumulation in the substantia nigra of both Parkin mutation-induced autosomal recessive juvenile PD cases and sporadic PD patients [119,120]. Lundius et al. [121] discovered that PSAP, as an endogenous ligand for GPR37, binds to GPR37 and promotes its translocation to the plasma membrane, increasing the density of GPR37 on the membrane. GM1 gangliosides are present in lipid rafts of the plasma membrane. The presence of PSAP facilitates the binding of GPR37 to GM1-enriched lipid rafts, forming a PSAP-GPR37-GM1 complex, which enhances cell viability. The results indicate that PSAP, GPR37, and GM1 constitute a signaling pathway that enhances cell survival through facilitating GPR37 translocation to the plasma membrane and subsequent integration into GM1-enriched lipid rafts. This finding reveals the potential roles of PSAP and GM1 in regulating the intracellular distribution of GPR37. They can protect dopaminergic neurons from PD-related toxic damage, providing new potential targets for the treatment of PD.

GD and PD are inextricably linked. The most significant common genetic risk factor for Parkinson’s disease is variants at the *Glucocerebrosidase (GBA)* gene locus, which encodes the enzyme glucocerebrosidase [122]. It was found that patients with GD and heterozygous carriers carrying mutations in the *GBA* gene developed PD at a higher frequency than the control population, suggesting that mutations in the *GBA* gene may be involved in the pathogenesis of PD. There is an interaction between α-Syn and GBA, and their levels are negatively correlated. Sap C, a coactivator of GBA, protects GBA from α-Syn inhibition, thus maintaining GBA activity. In the PD model, the activity of GBA was inhibited, and the decrease in the level of Sap C further exacerbated the decrease in GBA activity. By increasing the level of Sap C, the activity of GBA can be restored, which provides new ideas and potential intervention strategies for the treatment of PD [123]. Yap et al. [124] found that Sap C protects GCase from α-syn inhibition and restores GCase activity by dissociating α-syn from GCase in solution and lipid vesicles, which is critical for maintaining normal GCase function. Meanwhile, Sap C is a key cofactor in the degradation of GluCer by GCase, and its deficiency leads to the accumulation of GluCer, which in turn triggers GD symptoms. In addition, the control of GCase activity and the interaction between α-syn and GCase by Sap C could significantly impact disease progression, with its absence potentially being a PD risk factor.

**Figure 3:**
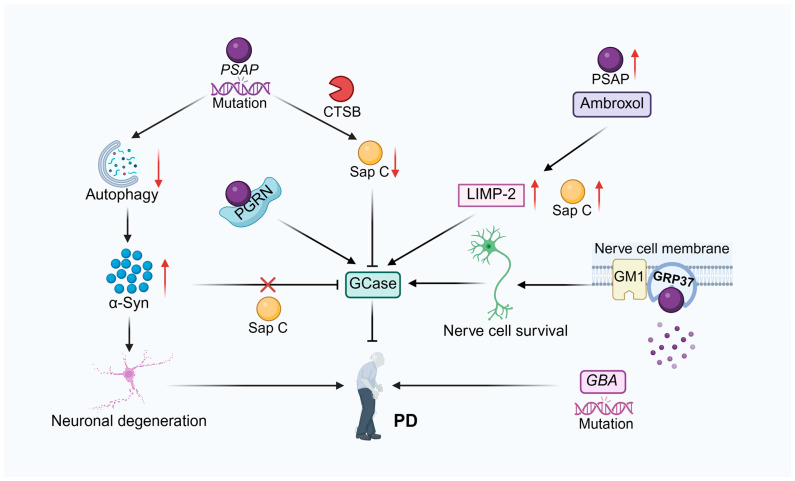
**PSAP in Parkinson’s disease (PD).** *PSAP* gene variants (e.g., rs4747203, rs885828) promote Parkinson’s disease (PD) progression by impairing autophagic flux, inducing α-synuclein (α-Syn) aggregation, and disrupting lysosomal function, leading to dopaminergic neuron degeneration. PSAP is processed by Cathepsin B (CSTB) to generate Sap C; thus, PSAP deficiency or mutations reduce levels of its derivative Sap C, suppressing glucocerebrosidase (GCase) activity and exacerbating pathological α-Syn accumulation. Conversely, PSAP overexpression or pharmacological interventions (e.g., ambroxol) upregulate Sap C and LIMP-2 levels, restore GCase activity and enhance α-Syn clearance, thereby inhibiting PD pathogenesis. PSAP interacts with progranulin (PGRN) in lysosomes, mutually enhancing their transport and activation, which further potentiates GCase activity. PSAP, as an endogenous ligand of GPR37, binds to GPR37 and facilitates its translocation to neuronal membranes, forming a PSAP-GPR37-GM1 complex within GM1-enriched lipid rafts to promote cell survival. GBA gene mutations significantly increase PD risk, while Sap C antagonizes α-Syn-mediated inhibition of GCase, restoring lysosomal function. These findings highlight the therapeutic potential of targeting PSAP-related pathways. Created with BioRender.com.

Drugs can treat PD associated with PSAP dysfunction. Mutations in the *GBA* gene, which codes for the enzyme glucocerebrosidase, are the most important common genetic contributor to the risk of developing PD [125,126]. Ambrosi et al. [127] found reduced GCase activity in fibroblasts from patients with PD with GBA1 mutations, and the drug ambroxol could correct this defect by increasing GCase activity and protein levels. In addition, ambroxol increased Sap C and Lysosomal Integral Membrane Protein 2 (LIMP-2) protein levels in all groups, but had little effect on Parkin levels. These findings suggest that ambroxol acts as a chemical chaperone that modulates lysosomal functions, in which increase of Sap C level is involved, providing a potential target for innovative therapeutic strategies for PD (Figure 3).

In summary, PSAP and its derivative Sap C play multifaceted roles in PD pathogenesis through regulating α-Syn homeostasis, GCase activity, and interactions with receptors GPR37/GPR37L1. Genetic evidence and studies utilizing cellular models strongly support the dysregulation of the PSAP pathway as a PD risk factor and a potential therapeutic target. However, it is important to note that the current understanding of PSAP’s neuroprotective mechanisms largely stems from neuronal models (e.g., PC12 cells, dopaminergic neuron-specific conditional knockouts) or analyses focused at the neuronal level. Astrocyte-expressed GPR37L1 has been identified as a critical receptor for PSAP and plays a central role in mediating protection against oxidative stress [39]. How PSAP, within the complex neuroinflammatory milieu of PD, modulates neuro-glia crosstalk–particularly via receptor signaling on astrocytes (and potentially microglia)–to influence dopaminergic neuron survival and resilience against α-Syn toxicity remains an under-explored area. To fully elucidate PSAP’s protective mechanisms in PD and develop more effective interventions, future research must integrate experimental tools specifically targeting glial cells (especially astrocytes), such as conditional genetic manipulations and pharmacological modulators, into existing PD models (both genetic and neurotoxin-induced). Furthermore, leveraging high-resolution spatial omics technologies (e.g., spatial transcriptomics, spatial proteomics) will be crucial for mapping the spatiotemporal dynamics of the PSAP-GPR37/GPR37L1 axis across different stages of PD pathology and brain regions (e.g., substantia nigra, striatum), and for understanding its overall impact on the neuro-glial network.

#### 3.2.2. Alzheimer’s Disease

Alzheimer’s disease (AD) is a neurodegenerative disorder characterized by behavioral and cognitive deficits [128] with the main pathological features being the accumulation of misfolded amyloid beta (Aβ) and neurofibrillary tangles (NFTs) [129,130]. Sharoar et al. [15] explored the role of PSAP through experiments in different mouse models of AD and brain samples from AD patients to investigate the role of PASP and its derived saposins in AD. It was found that lysosomes containing saposins and lysosome-associated membrane protein (LAMP1) accumulate in dystrophic neurites (DNs) surrounding Aβ plaques during the early stages of AD, but lysosomal hydrolases (e.g., CTSB and CTSBD) were not detected in these early DNs, implying that these lysosomes may be malfunctioning lysosomes. As AD pathology progresses, accumulation of Sap C in lysosomes may be associated with lysosomal dysfunction. Increased Sap C may lead to increased lysosomal membrane permeability (LMP), which in turn triggers lysosomal damage and cell death, exacerbating the pathologic course of AD. In cerebrospinal fluid (CSF) from patients with AD, Heywood et al. [131] found that PSAP expression levels were approximately 1.7-fold higher than in controls. Another study identified PSAP as a potential serum biomarker for AD. PSAP was analyzed by liquid chromatography-tandem mass spectrometry (LC-MS/MS) technique in low-molecular-weight serum proteins of patients with mild cognitive impairment (MCI) and patients with mild AD. The study utilized longitudinal samples from the same individuals, and PSAP expression was found that PSAP expression was increased in samples collected after progression from MCI to mild AD (i.e., post-cognitive decline) compared to their pre-decline baseline [132]. Aberrant splicing and point mutations in the human presenilin genes, PSEN1 and PSEN2, have been associated with familial AD, and the study found increased PSAP expression following splicing disruption of the *PSEN1* and *PSEN2* genes in *zebrafish* [133]. Mechanistically, PS18 inhibited GSK-3β/α activity via activation of the PI3K/Akt signaling pathway, which facilitated the non-amyloidogenic processing of AβPP and reduced Aβ_1–42_ production. Additionally, PS18 demonstrated neuroprotective effects by suppressing the Caspase pathway, upregulating Bcl-2 expression, downregulating BAX, and attenuating mitochondrial damage and Caspase-3 activation [134]. In summary, PS18 counteracted Aβ_1–42_-induced neurotoxicity through multi-pathway synergy, restored balanced hippocampal neurogenesis, and improved cognitive function, offering novel insights for the therapy against AD.

**Figure 4:**
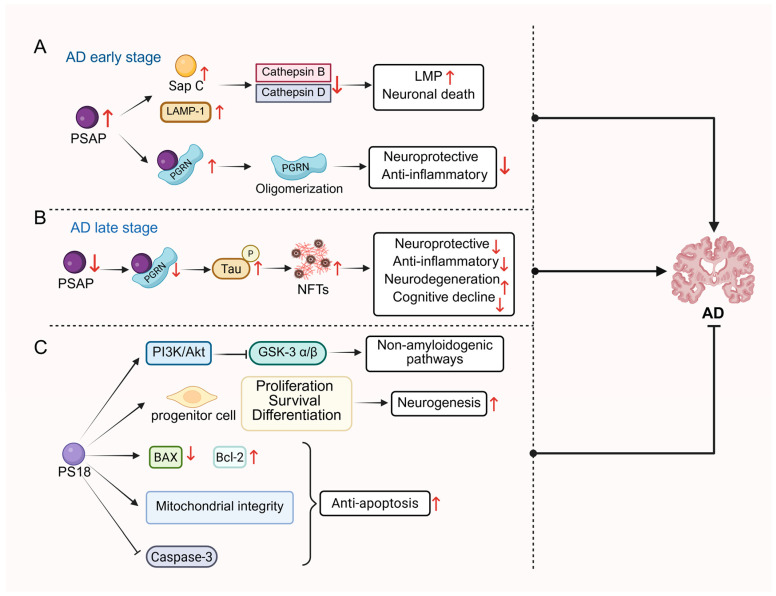
**PSAP in Alzheimer’s disease (AD).** PSAP has multiple roles in Alzheimer’s disease (AD) from early Aβ pathology to late tau-mediated neurodegeneration. (**A**) In early stages, PSAP accumulates in dystrophic neurites surrounding Aβ plaques, where lysosomes enriched with Sap C and LAMP1 exhibit impaired hydrolase activity (e.g., absence of cathepsin-B/D), leading to lysosomal membrane permeabilization (LMP) and neuronal death. Concurrently, PSAP interacts with progranulin (PGRN) in neurons and microglia, promoting PGRN oligomerization and its pathological sequestration within Aβ plaques, thereby diminishing the neuroprotective and anti-inflammatory functions of PGRN. (**B**) As AD progresses to tau-dominated stages, PSAP expression declines in neurons harboring neurofibrillary tangles (NFTs), disrupting its interaction with PGRN and exacerbating tau hyperphosphorylation and aggregation, which ultimately promotes NFT formation, compromises PGRN-mediated neuroprotection and anti-inflammatory functions, and accelerates neurodegeneration and cognitive decline. (**C**) Therapeutic intervention with the PSAP-derived peptide PS18 counteracts Aβ toxicity by activating the PI3K/Akt pathway to suppress GSK-3β/α activity, shifting amyloid precursor protein (APP) processing toward non-amyloidogenic pathways, and enhancing hippocampal neurogenesis through regulation of progenitor cell proliferation, survival, and differentiation. PS18 further mitigates apoptosis by modulating Bcl-2/BAX balance, preserving mitochondrial integrity, and inhibiting caspase-3 activation. PSAP emerges as a critical regulator of lysosomal dysfunction, protein aggregation, and neuroinflammation, with its dual roles in early Aβ pathology and late tauopathy, highlighting its potential as a multi-target therapeutic candidate for AD. Created with BioRender.com.

PGRN is a protein encoded by the *GRN* gene which participates in diverse physiological and pathological processes including inflammation regulation, wound healing and tumorigenesis. It can be transported to lysosomes independently of the Sortilin pathway through interaction with PSAP and maintains cellular homeostasis by regulating lysosomal enzyme activities [135]. The interaction between PGRN and PSAP, which leads to PGRN aggregation in Aβ plaques, has been shown to affect their biological activity. This aggregation may limit the neuroprotective and anti-inflammatory effects of PGRN and thus play a role in the early pathology of AD. In AD cases, PSAP expression levels were increased and co-localized with PGRN in neuronal cells and microglial cells, while co-localization of the two was also observed in Aβ plaques [136]. Overexpression of PSAP was able to increase the aggregation state of PGRN, whereas lack of PSAP may have led to impaired transport of PGRN to the lysosome but increased the circulating levels of PGRN. These findings suggest that PSAP critically modulates the aggregation and transportation of PGRN, thereby influencing its role in AD pathology [136]. In another study, the researchers found in different types of cell lines that PSAP expression levels were significantly correlated with plasma PGRN levels, and that both reduction and overexpression of PSAP resulted in significant increases in extracellular PGRN levels, but that PGRN monomers were increased when PSAP was reduced, whereas PGRN oligomerization was increased when PSAP was overexpressed. PSAP interacts with PGRN extracellularly, and direct binding of the two can promote PGRN oligomerization. This oligomerized state may affect the biological activity of PGRN, causing it to be deposited in Aβ plaques and limiting its ability to be cleared by microglial cells, thereby exacerbating the pathological process of AD. In a cell model carrying a mutation in the *GRN* (Progranulin gene), the regulatory effect of PSAP was able to restore PGRN levels, suggesting that PSAP is a novel regulator of PGRN levels and oligomerization, and a potential target for treating frontotemporal dementia (FTD) and other neurodegenerative diseases [137]. In contrast, the interaction of PSAP with PGRN has different effects in the late tau pathologic stage of AD. Mendsaikhan and his colleagues [108] found that PSAP expression was significantly reduced in NFTs in the hippocampus and middle temporal gyrus regions of the brain in both controls and patients with AD, and that the fluorescence intensity of PSAP was significantly reduced in NFTs compared to neighboring neurons that did not form tangles, and that this difference was more pronounced in AD cases. In addition, the investigators confirmed the interaction of PSAP with PGRN in normal neurons in human brain tissue sections, but this interaction was significantly reduced in NFTs. These results suggest that PSAP deficiency may promote the formation of NFTs in conjunction with the reduction of PGRN, and that the interaction between PSAP and PGRN may be inhibited during the pathologic process of NFTs, which in turn exacerbates the pathologic process of AD. In conclusion, PGRN and PSAP interact to play critical roles in different pathological stages of AD. In the early stage, their interaction affects the activity and aggregation of PGRN, contributing to Aβ-related pathology. Regulation of PGRN levels, monomers and oligomers by PSAP also exacerbates AD pathology. In the late tau pathological stage, the reduced expression of PSAP and the attenuated interaction of PSAP with PGRN promotes the formation of NFTs and worsens the condition of AD. Thus, PSAP may serve as a potential target for treating AD-related neurodegenerative diseases (Figure 4).

### 3.3. Atherosclerosis

Atherosclerosis (AS), the most common form of cardiovascular disease, is characterized by vascular wall accumulation of oxidized low-density lipoprotein (ox-LDL), increased atherosclerotic inflammation, and endothelial cell (EC) and vascular smooth muscle cell (VSMC) dysfunction, ultimately leading to plaque formation [138]. As a lipid-induced progressive inflammatory disease of the arterial vascular wall, AS can lead to serious complications such as myocardial infarction and stroke [139]. Accumulation of low-density lipoproteins and activation of endothelial cells, which promote the recruitment of inflammatory cells, are involved in the pathogenesis of AS. As monocytes, macrophages, and other cells accumulate within the plaque, their nutrient availability decreases and cell death increases, leading to large necrotic nuclei in advanced atherosclerotic plaques [140]. In advanced atherosclerotic plaques, pro-inflammatory macrophages secrete matrix-degrading enzymes that thin the fibrous cap, leading to plaque instability and rupture [141]. Van Leent and his colleagues found PSAP to be coexpressed with inflammatory markers, namely APOE, APOC1, CCL2, CTSB, CTSD, and MMP9 [16], which is similar to previous observations by Fernandez et al. [142]. In addition, they analyzed subpopulations of macrophages in human AS plaques using single cell RNA sequencing and found that a high level of PSAP expression in these cells was associated with inflammation. In all eukaryotic cells, the mTOR signaling network not only underlies the balancing of anabolic and catabolic pathways in response to trophic status but also acts as a central modulator of inflammatory responses in immune cells [143]. Van Leent et al. [16] found that inhibition of the mTOR and S6K1 signaling pathways significantly reduced the inflammatory response in AS plaques in APOE^-/-^ mice by using nanobiotics with mTOR inhibitors (e.g., rapamycin) and S6K1 inhibitors (e.g., PF-4708671). In addition, they found by transcriptome analysis that PSAP expression was positively correlated with the activation level of mTOR signaling pathway and that silencing of PSAP inhibited glycolysis and oxidative phosphorylation in macrophages. In the APOE^-/-^ mouse model, mice transplanted with PSAP^-/-^ bone marrow exhibited reduced AS plaque volume and inflammatory cell infiltration. These results suggest that PSAP, which could be induced by mTOR and S6K1, is an important mediator of macrophage inflammatory effects in AS plaques. Therefore, targeting PSAP or its individual saposin structural domains may be one of the potential strategies for the treatment of AS.

Macrophages in the arterial endothelium can remove lipoproteins and eventually become foam cells that secrete inflammatory molecules and promote inflammation [144]. Notably, this lipid overload and pro-inflammatory transformation also impair the ability of macrophages to perform efferocytosis, a critical process for maintaining plaque stability. Clearance of apoptotic cells (AC) from the necrotic core of plaques is mediated by efferocytes (especially macrophages) in a process known as efferocytosis, which is essential for tissue homeostasis [145,146]. In advanced AS lesions, impaired efferocytosis contributes to plaque necrosis and fibrous cap thinning, which can ultimately trigger plaque rupture, thrombosis, and myocardial infarction [145,147]. This process involves PSAP-GPR37-mediated activation of ERK signaling, which upregulates c-fos expression. (an important component of AP-1 transcription complex), leading to enhanced transcription of a key macrophage efferocytosis receptor Tim4, thereby promoting apoptotic cell recognition and sustained clearance [148]. In APOE^-/-^ mice, Effero-EVs reduced necrosis of atherosclerotic plaques, decreased lesional inflammation, and increased collagen deposition [148].

**Figure 5:**
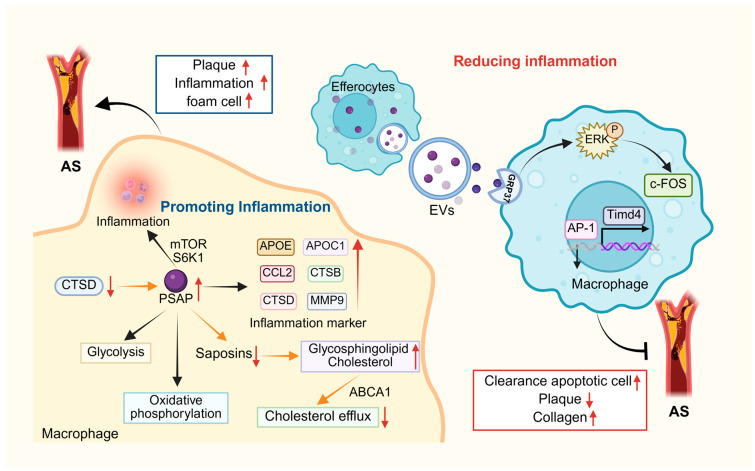
**PSAP in Atherosclerosis (AS).** PSAP regulates macrophage function in atherosclerosis (AS) by a dual mechanism. (Left) Intracellularly, PSAP is processed into mature saposins by Cathepsin D (CTSD) to regulate sphingolipid degradation; its functional abnormalities (e.g., reduced CTSD activity) lead to the accumulation of glycosphingolipids and cholesterol in lysosomes, impeding ABCA1-mediated cholesterol efflux and accelerating foam cell formation. In addition, PSAP is co-expressed with APOE, APOC1, CCL2, CTSB, CTSD and MMP9, which play important roles in plaque inflammation. Furthermore, PSAP promotes macrophage inflammation via the mTOR/S6K1 pathway, and inhibition of mTOR/S6K1 reduces inflammatory responses and plaque volume in APOE^-/-^ mice, while PSAP deficiency suppresses macrophage glycolysis and oxidative phosphorylation to inhibit inflammatory activation of macrophages. (Right) Extracellularly, PSAP^+^ efferocytic extracellular vesicles (Effero-EVs) released by efferocytes bind to GPR37 on macrophages, activating ERK signaling and increasing c-Fos expression, which upregulates Tim4 to enhance apoptotic cell clearance. This process reduces plaque necrosis and increases collagen deposition. Advanced plaques with impaired efferocytosis exhibit necrotic core expansion and thin fibrous caps. Targeting PSAP may stabilize plaques by balancing inflammatory and efferocytic pathways. Created with BioRender.com.

As mentioned above, PSAP modulates macrophage function and plaque stability in atherosclerosis through multidimensional mechanisms. At the metabolic level, as a downstream effector of the mTOR-S6K1 signaling pathway, PSAP drives reprogramming of glycolysis and oxidative phosphorylation in macrophages, amplifying inflammatory activity and accelerating plaque progression; targeting this axis downregulates PSAP expression and suppresses metabolism-dependent inflammation [16]. In lipid clearance, CTSD-mediated processing of PSAP into mature saposins regulates sphingolipid degradation, and its dysfunction (e.g., due to reduced CTSD activity) causes glycosphingolipid and cholesterol accumulation in lysosomes, impairing ABCA1-mediated cholesterol efflux and promoting foam cell formation [149]. For inflammation resolution, PSAP-enriched extracellular vesicles (EVs) released by efferocytes enhance macrophage efferocytosis via the GPR37-ERK-AP1 axis, upregulating Tim4 receptor expression to polarize macrophages toward reparative M2 phenotypes, thereby reducing plaque necrotic cores and increasing collagen deposition [148]. These findings highlight PSAP’s potential as a pleiotropic therapeutic target: PSAP-specific silencing using siRNA-loaded nanoparticles concurrently suppresses metabolic inflammation and lipid accumulation; enhancing CTSD activity restores lysosomal lipid homeostasis; while exogenous PSAP-EVs or GPR37 agonists selectively rescue efferocytosis defects. Combinatorial approaches with existing therapies (e.g., S1P receptor modulators) may synergistically optimize plaque stabilization [16]. Future studies should further elucidate the dynamic regulatory network of PSAP within plaque microenvironments, particularly how its subcellular translocation influences metabolic-inflammatory-efferocytic functional switching in macrophages. Concurrently, developing spatiotemporally precise PSAP-targeted delivery systems (e.g., conditionally activated nanocarriers) remains crucial to circumvent potential off-target effects on systemic sphingolipid metabolism. Integrating multi-omics technologies with live imaging to evaluate combined PSAP-targeting regimens (e.g., mTOR inhibitors with GPR37 agonists) for advanced plaque regression may pioneer new avenues for precision intervention in atherosclerosis (Figure 5).

### 3.4. Cancer

#### 3.4.1. Breast Cancer

**Figure 6:**
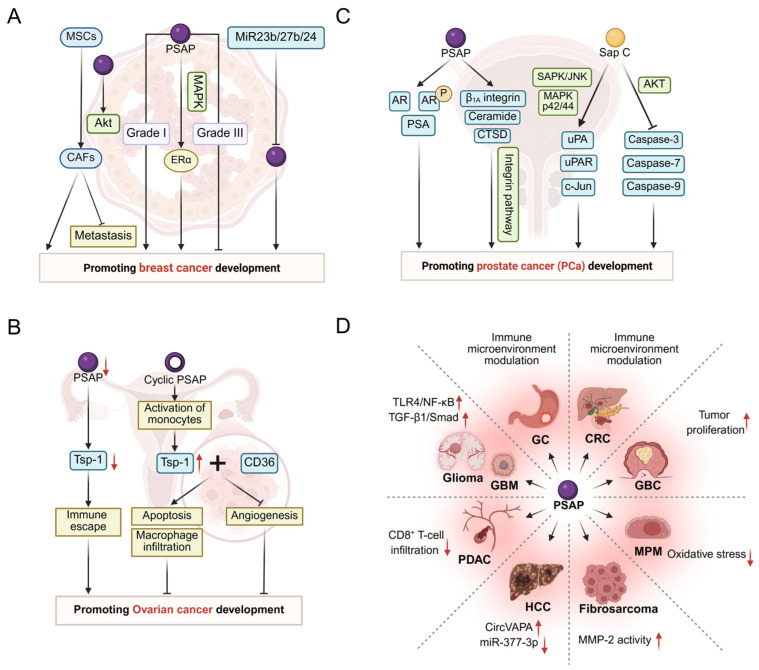
**PSAP and cancers.** PSAP regulates tumor progression through diverse signaling pathways and molecular interactions. (**A**) In breast cancer, PSAP upregulates ERα expression via the MAPK signaling pathway, enhancing its intranuclear translocation and transcriptional activity to drive estrogen-dependent breast cancer growth. Cancer-associated fibroblasts (CAFs), differentiated under rigid extracellular matrix (ECM) conditions, secrete PSAP to activate the Akt pathway, supporting breast cancer cell proliferation while inhibiting metastasis. Additionally, the miR-23b/27b/24 cluster promotes aggressive breast cancer metastasis by directly suppressing PSAP expression. PSAP exhibits dual roles in breast cancer progression: promoting tumorigenesis in grade I tumors and suppressing metastasis in grade III tumors, highlighting its context-dependent regulatory function. (**B**) In ovarian cancer, inhibition of PSAP (prosaposin) by the tumor microenvironment leads to decreased secretion of thrombospondin-1 (TSP-1), enabling tumors to achieve immune evasion. The therapeutic cyclic PSAP peptide restores TSP-1 production by activating bone marrow-derived monocytes. TSP-1 binding to CD36 induces apoptosis, inhibits angiogenesis, inhibits angiogenesis, promotes macrophage infiltration, significantly suppressing tumor growth and metastasis. (**C**) Role of PSAP in other cancers. In prostate cancer (PCa), PSAP upregulates activity of hormone receptor (AR) and prostate-specific antigen (PSA) expression and phosphorylation, and promotes tumor growth, migration, and invasion through the integrin pathway (via β_1A_-integrin, Cathepsin D [CTSD], and ceramide). Sap C further stimulates urokinase-type plasminogen activator (uPA)/uPA receptor (uPAR) and c-Jun, activates the p42/44 mitogen-activated protein kinase (MAPK) and stress-activated protein kinase/c-Jun N-terminal kinase (SAPK/JNK) pathways, and suppresses apoptosis via Akt-mediated inhibition of caspase-3/7/9, thereby stimulating proliferation, migration, and invasion of PCa cells. (**D**) In gliomas and glioblastoma (GBM), PSAP promotes tumor invasion and epithelial–mesenchymal transition (EMT) through TLR4/NF-κB and TGF-β1/Smad pathways. In gastric cancer (GC) and colorectal cancer (CRC), high PSAP expression correlates with advanced tumor stages and poor prognosis, potentially promoting progression via immune microenvironment modulation. In gallbladder cancer (GBC), PSAP upregulation promotes proliferation and serves as an early diagnostic biomarker. In hepatocellular carcinoma (HCC), PSAP drives tumor proliferation via the circVAPA/miR-377-3p axis. In pancreatic ductal adenocarcinoma (PDAC), PSAP suppresses CD8^+^ T-cell infiltration to accelerate progression. In malignant pleural mesothelioma (MPM), PSAP supports tumor survival by resisting oxidative stress. In fibrosarcoma, PSAP enhances Matrix Metalloproteinase-2 (MMP-2) activity to promote invasion. Created with BioRender.com.

Breast cancer is the number one cause of cancer deaths in women [150]. Estrogen 17β-estradiol (E2) is an important female sex steroid hormone that is crucial in the development and progression of breast cancer [151]. Studies have shown that E2 induces and promotes breast cancer, a process mediated primarily by estrogen receptor α (ER α) [152]. PSAP upregulates ERα expression, enhances its nuclear translocation and transcriptional activity through MAPK signaling, and significantly accelerates breast cancer cell growth in vitro and in vivo [17]. In a study by Meijer et al. [153], Elevated PSAP mRNA was significantly associated with shorter progression-free (PFS) survival in patients with estrogen receptor (ER)-positive breast cancer treated with tamoxifen. Another study showed that in breast and ovarian cancer cell lines from human, there is specific mannose-6-phosphate (M6P) receptor-independent interaction between procathepsin D (proCath-D) and PSAP, occurring in both the intracellular space and secretory media. PSAP can interact with proCath-D. Inhibition of low-density lipoprotein receptor-associated protein (LRP)-mediated endocytosis using receptor-associated protein (RAP) blocked PSAP endocytosis in rat fibroblasts but had no effect on the M6P-independent endocytosis of proCath-D in MDA-MB231 cells. This indicates that the endocytosis of proCath-D is independent of PSAP and occurs through a different process from that of the M6P receptors and LRP. Although the exact role of the interaction between PSAP and proCath-D in cancer cells remains unclear, it may be involved in lysosomal targeting, protease clearance, or the growth-stimulating action of these two proteins [154]. Previous studies have demonstrated that Cancer-associated fibroblasts (CAFs) are widely present in the microenvironment of almost all tumors and have a significant impact on tumor progression [155]. Ishihara et al. [156,157] identified cancer-associated fibroblasts (CAFs) secrete PSAP in the rigid extracellular matrix (ECM) microenvironment. ECM stiffness induces mesenchymal stem cells (MSCs) to differentiate into CAFs via mechanosignaling, promoting breast cancer cell proliferation/survival but inhibiting metastasis, partly through PSAP-activated Akt signaling. PSAP expression associates with poor prognosis in grade I breast cancer but improved prognosis in grade III. To inhibit breast cancer progression, Ell et al. [158] found that expression of the miR-23b/27b/24 cluster correlated with metastatic potential in mouse and human breast cancer cell lines and was upregulated in metastatic lung lesions in patients with breast cancer. Gene expression analysis revealed that *PSAP* is a direct target of miR-24 and miR-27b, and its expression was negatively correlated with metastatic progression in patients with breast cancer. Significantly, the miR-23b/27b/24 cluster promotes metastasis by directly suppressing PSAP. Ectopic PSAP expression inhibits metastasis in aggressive breast cancer cells, even those overexpressing miR-23b/27b/24. (Figure 6).

#### 3.4.2. Ovarian Cancer

Ovarian cancer is the deadliest gynecologic malignancy and fourth leading cause of cancer death in women, with poor survival in advanced stages [159]. In high-grade plasmacytoid ovarian cancer, the tumor microenvironment suppresses PSAP expression during progression, reducing secretion of anti-tumor protein thrombospondin-1 (TSP-1) and enabling immune evasion. However, the expression of TSP-1 receptor CD36 is preserved or even elevated in tumor cells, which may be due to the fatty acid metabolism mediated by CD36 supporting tumor growth in metastatic sites such as omentum. At the same time, the high expression of CD36 also enables TSP-1 to bind with it specifically, which triggers downstream apoptotic signaling pathways, promoting tumor cell apoptosis. To treat metastatic ovarian cancer, a prosaposin-derived cyclic peptide was tested in patient-derived xenograft (PDX) models. Intraperitoneal injection of cyclic PSAP peptide activated bone marrow-derived monocytes, elevating TSP-1 expression. This restored TSP-1’s anti-tumor effects: binding tumor CD36 to induce apoptosis, inhibited angiogenesis, and promoted macrophage infiltration, significantly suppressing tumor growth and metastasis without observed toxicity. These results suggest that cyclic PSAP peptide reactivates the anti-tumor mechanism in the tumor microenvironment by stimulating the expression of TSP-1, providing a new and efficient therapeutic strategy for the treatment of metastatic ovarian cancer [160] (Figure 6).

#### 3.4.3. Prostate Cancer

Prostate cancer (PCa) represents the most frequently diagnosed non-skin cancer among males in western populations and ranks as the second most common cause of cancer-related mortality in men within the United States [161]. PCa can be divided into Localized Prostate Cancer (LPC) and Metastatic Prostate Cancer (MPC) according to the stage of the disease, and into Castration-Sensitive Prostate Cancer (CSPC) and Castration-Resistant Prostate Cancer (CRPC) according to hormone therapy sensitivity [162]. Koochekpour identified PSAP as an androgen regulator that promotes cancer calls (LNCaP) growth, migration, and invasion via upregulation of androgen receptors (ARs) and their target genes (e.g., Prostate-Specific Antigen, PSA) [18]. They identified overexpression of PSAP in androgen-independent (AI) prostate LNCaP through cloning and sequencing, and demonstrated its association with enhanced cell proliferation, migration, invasion, and anti-apoptotic effects, suggesting a potential role for PSAP in PCa carcinogenesis or progression [163]. Interestingly, Hu et al. [164] stably down-regulated PSAP expression in metastatic LNCaP by RNA interference techniques and found that intracellular ceramide levels, CTSD and β_1A_-integrin expression were decreased, and attenuation of “inside-out” integrin-signaling pathway suggested the involvement of PSAP in PCa invasion. Serum PSAP levels were particularly lower in primary confined PCa and higher in patients with metastatic castrate-resistant prostate cancer (mCRPCa) compared with normal individuals. The findings suggest that PSAP has the potential to be a discriminator between primary and advanced PCa [165].

Researchers also found that not only PSAP, but also Sap C could exhibit androgen agonistic effects in LNCaP cells by increasing AR mRNA and protein expression, nuclear AR content and its phosphorylation status, as well as PSA mRNA and protein expression [166]. The study reveals a bidirectional regulation between PSAP/Sap C and AR signaling in PCa. Androgens (e.g., dihydrotestosterone, DHT) directly activate the PSAP promoter via a hormone-responsive element (HRE), increasing PSAP expression. In turn, PSAP and Sap C enhance AR activity in a ligand-independent manner by upregulating AR expression, promoting its nuclear translocation and inducing tyrosine phosphorylation. This crosstalk is further amplified by serum-derived factors (e.g., growth factors and hormones), which synergize with DHT to stimulate PSAP transcription, as shown by serum-induced luciferase activity in both androgen-sensitive and androgen-independent (AI) PCa cells. Furthermore, conditioned media from prostate stromal cells (PrSt) and bone fibroblasts (MSF), which contain PSAP/Sap C being secreted by the above cells, can enhance PSAP promoter activity in PCa cells. This indicates that PrSt/MSF-derived PSAP/Sap C stimulates PSAP transcription within tumor cells through paracrine signaling, possibly by enhancing AR activity, which forms a feed forward loop involving PSAP/Sap C and AR signaling in PCa cells [167]. These findings suggest that the PSAP/AR axis not only fuels early androgen-dependent prostate carcinogenesis but also supports AI progression by maintaining AR activity in a hormone-deprived tumor microenvironment. Meanwhile, Sap C, as a multipotential regulator of PCa and stromal cells, can upregulate the expression of urokinase-type fibrinogen activator (uPA) and its receptor (uPAR) and the immediate-early gene c-Jun in a cell-type-specific manner and stimulate cell proliferation, migration, and invasion in prostate stromal and carcinoma cells, as well as activate p42/44 MAPK and Stress-Activated Protein Kinase/c-Jun N-Terminal Kinase (SAPK/JNK) pathways [168]. Lee et al. [169] found that Sap C activates Akt under serum starvation, suppresses Caspase-3/7/9 and reduces the cleaved nuclear substrate of Caspase-3 in PCa cells, acting as a survival factor. In addition, Sap C reduced cell growth inhibition, activity of Caspase-3/7 and apoptotic cell death induced by etoposide. The above studies suggest that Sap C and PSAP serve as a pro-mitotic, survival and anti-apoptotic factors to facilitate the initiation and progression of PCa (Figure 6).

Collectively, PSAP and its derivative Sap C play pivotal roles in PCa initiation, progression, and transition to CRPC by engaging in positive feedback regulation of the AR signaling pathway (including upregulating AR expression, promoting its nuclear translocation and phosphorylation), and activating downstream pro-survival (e.g., PI3K/Akt), pro-migration/invasion (e.g., MAPK/JNK, uPA/uPAR) pathways. However, the current mechanistic understanding of PSAP/Sap C-driven oncogenesis is predominantly derived from studies utilizing classical cell lines (e.g., androgen-sensitive LNCaP) in vitro and xenograft models in immunocompromised mice (e.g., nude mice). While these models have successfully uncovered the core interaction between PSAP/Sap C and AR and their capacity to activate key intracellular signaling cascades, they suffer from significant limitations: they inadequately recapitulate the complex and dynamically evolving TME found in humans, particularly failing to effectively model the intricate stromal interactions (e.g., communication with fibroblasts, immune cells), tumor heterogeneity, and adaptive responses under therapeutic pressure characteristic of the post-androgen deprivation therapy CRPC stage. To more accurately assess the true contribution of PSAP/Sap C to PCa disease progression (especially metastasis and therapeutic resistance) and validate its translational potential as a therapeutic target, future research must leverage more advanced and physiologically relevant model systems. These include employing patient-derived organoids that preserve primary tumor heterogeneity and stromal elements; establishing humanized immune system mouse models to incorporate functional human immune components; and developing orthotopic transplantation models mimicking CRPC microenvironments (e.g., bone metastatic niche). Utilizing such models will not only provide more reliable validation of PSAP/Sap C function in advanced PCa but also enable in-depth investigation into the specific molecular mechanisms by which they mediate bidirectional communication between tumor cells and key stromal partners (e.g., cancer-associated fibroblasts CAFs, tumor-associated macrophages TAMs) within the TME. This knowledge is essential for rationally developing novel therapeutic strategies targeting the PSAP/Sap C axis, potentially in combination with androgen deprivation therapy or immunotherapy.

#### 3.4.4. Other Cancers

##### Gliomas and Glioblastoma

Gliomas are the most prevalent primary CNS tumors with poor prognosis (<15 months survival) [170]. Recent studies have shown that gliomas can promote self-renewal, angiogenesis, and invasion through the release of a variety of autocrine or paracrine secreted proteins [171]. PSAP overexpression in gliomas activates TLR4/NF-κB signaling, driving inflammatory factor release and glioma stem cell growth. TLR4 inhibitor TAK-242 blocks PSAP-induced proliferation, identifying PSAP as a therapeutic target [19].

In the central nervous system, glioblastoma (GBM) is the most aggressive and deadly malignant tumor [172]. The mesenchymal subtype of GBM is the most aggressive. According to Verhaak’s molecular typing, GBM can be categorized into preneuronal, neural, classic and mesenchymal types [173]. Among these four subtypes, mesenchymal GBM is the most aggressive, and has the highest recurrence and drug resistance rates with the worst prognosis [174]. Jiang et al. [175] demonstrated through in vitro and in vivo experiments that PSAP promotes the invasive and epithelial–mesenchymal transition (EMT)-like process of GBM, and that this effect was mainly achieved by activating the TGF-β1/Smad signaling pathway. Thus, it suggests that PSAP may be a potential target for mesenchymal GBM therapy (Figure 6).

##### Gastric Cancer

Gastric cancer (GC) has a high global mortality rate with advanced-stage 5-year survival < 25%. The tumor immune microenvironment critically influences GC progression, where distinct immune subtypes show varying prognoses and treatment responses s [176]. Wen et al. [20] analyzed data from the Cancer Genome Atlas (TCGA), revealing significantly elevated PSAP mRNA in GC tissues versus normal tissues. High PSAP expression correlated with advanced tumor stage and reduced overall survival, indicating poorer prognosis. Using the TISIDB database, PSAP significantly associated with three immunomodulatory genes, immune checkpoint inhibitor PDCD1, pro-tumorigenic TGFB1, and pro-tumorigenic CSF1R. A risk model based on these three genes predicted prognosis and immunotherapy efficacy in stomach adenocarcinoma (STAD) patients. Stratifying patients into high/low-risk groups by risk scores, the model’s accuracy was validated via Kaplan–Meier analysis and Cox regression. The researchers noted that the expression and function of PSAP in STAD may have an important role in facilitating tumor proliferation, tumor formation and metastasis, and may also be a potential biomarker and therapeutic target for clinical treatment and prognostic assessment of GC (Figure 6).

##### Colorectal Cancer

Colorectal cancer (CRC) ranks among the most prevalent cancers [177] and is a leading cause of cancer-related mortality [178]. Using quantitative label-free proteomics analysis, researchers have found that PSAP is more highly expressed in CRC subtypes linked to poorer prognosis, including consensus molecular subtype 4 (CMS4), Colorectal Cancer Intrinsic Subtype B (CRIS-B), and Stem-like subtype, compared with other subtypes. In experimental models, PSAP expression was also elevated in highly metastatic cell lines compared to low-metastatic cell lines. Additionally, high PSAP expression is more common in CRC patients with dMMR (deficient mismatch repair), CIMP+ (CpG island methylator phenotype) status, and BRAF mutations, which are molecular features that collectively enhance tumor aggressiveness and cause poor prognosis: dMMR leads to mutation accumulation due to defective DNA repair; CIMP+ silences tumor suppressor genes via hypermethylation; and BRAF mutations activate pro-proliferative signaling pathways. The high expression of PSAP is associated with reduced overall, progression-free, and disease-specific survival in stages II/III patients. Researchers developed the SEC6 risk-scoring algorithm combining PSAP with IGFBP3, CD109, LTBP1, BMP1, and NPC2 expression to stratify patients and guide personalized treatment, confirming PSAP’s value in CRC prognosis and chemotherapy prediction [21] (Figure 6).

##### Gallbladder Cancer

Gallbladder cancer (GBC) is a rare but highly aggressive malignancy with high lethality, ranking as the fifth most common gastrointestinal cancer and leading biliary tract malignancy [179]. Sahasrabuddhe et al. [22] performed iTRAQ-based quantitative proteomics profiling of GBC, identifying PSAP expression increased 2.7-fold in tumor tissues versus adjacent non-tumor tissues. Tissue microarrays and immunohistochemical staining validated strong PSAP positivity in 83% of GBC samples, significantly differing from non-tumor tissues. PSAP upregulation may confer survival advantages to GBC and, due to its secretory nature in bodily fluids, it may serve as an early diagnostic biomarker for GBC, potentially contributing to early detection and treatment of GBC (Figure 6).

##### Hepatocellular Carcinoma

The systemic disease known as metabolic dysfunction-associated steatotic liver disease (MASLD) is identified by insulin resistance and lipotoxicity [180], is a major global health burden that can progress to hepatocellular carcinoma (HCC) [181,182,183], often diagnosed at advanced stages [184]. HCC ranks as the third leading cause of cancer death worldwide [185]. Liu et al. [23] identified upregulation of circular RNA circVAPA in HCC cell lines; its knockdown suppressed proliferation. Mechanistically, circVAPA promotes PSAP expression by inhibiting miR-377-3p. Overexpressing PSAP partially rescues proliferation in circVAPA-silenced cells, confirming PSAP as essential for circVAPA-driven proliferation. These findings reveal a novel mechanism by which circVAPA promotes HCC cell proliferation through the miR-377-3p/PSAP axis and provide potential biomarkers and therapeutic targets aimed at treating HCC (Figure 6).

##### Pancreatic Ductal Adenocarcinoma

An important reason for the poor prognosis of patients with pancreatic ductal adenocarcinoma (PDAC) may be its unique tumor microenvironment (TME), an environment characterized by dense stroma and low infiltration of anti-tumor T-cells [186]. Through glycoproteomics and tandem mass spectrometry tagging (TMT) labeling techniques, investigators identified the secreted protein PSAP in the culture medium of three human PDAC cell lines (BxPC-3, PANC-1, MIA PaCa-2) and found that it was highly expressed in multiple PDAC cell lines. Clinical analysis linked high PSAP to larger tumor volume, reduced CD8^+^ T-cell infiltration, and worse prognosis. In vitro, PSAP decreased CD8^+^ T-cell proportions in peripheral blood mononuclear cells. In vivo, shRNA-mediated PSAP knockdown in murine PKCY cells increased intratumoral CD8^+^ T-cells and reduced tumor volume, suggesting that PSAP may promote the progression of PDAC by inhibiting CD8^+^ T-cells and PSAP modulation may be a potential new strategy for immunotherapy of PDAC [24] (Figure 6).

##### Malignant Pleural Mesothelioma

Malignant pleural mesothelioma (MPM) accounts for less than 1% of all cancers, but its incidence is expected to continue to grow until at least 2030. Most patients are diagnosed at advanced stages due to diagnostic challenges, exhibiting median survival usually less than 12 months [187]. Through proteomic analysis, Lacerenza et al. [25] found that PSAP is one of the highly upregulated proteins secreted by MPM cells, suggesting that it could be crucial in defending cancer cells against oxidative stress and assisting them in escaping apoptosis, thus aiding in the advancement of MPM. Comparing the MPM cell line with the non-malignant mesothelial cell line, the expression level of PSAP was significantly increased in MPM cells. In addition, the researchers detected significantly higher levels of PSAP in the serum of MPM patients than in controls, revealing that PSAP may serve as a potential biomarker for MPM. Although ROC analysis showed limited diagnostic specificity/sensitivity for PSAP alone, combining PSAP with Quiescin Q6 Sulfhydryl Oxidase 1 (QSOX1) significantly improved diagnostic accuracy, supporting its potential as a biomarker in multiprotein panels (Figure 6).

##### Fibrosarcoma

Previous studies have demonstrated that tissue factor pathway inhibitor-2 (TFPI-2) is associated with fibrosarcoma [188] and that downregulation of TFPI-2 is associated with tumor growth/metastasis [189]. Researchers identified PSAP as a new interaction partner of TFPI-2 by yeast two-hybrid screen and later verified the interaction between the second Kunitz-type domain (KD2) of TFPI-2 and the C-terminus of PSAP. It was found that PSAP did not affect TFPI-2’s serine protease inhibitory function. TFPI-2 significantly inhibited PSAP’s invasion- and migration-promoting effects in human fibrosarcoma HT1080 cells. Additionally, TFPI-2 blocked PSAP-induced enhancement of matrix metalloproteinase-2 (MMP-2) activity, which is closely linked to tumor cell invasive/migratory capabilities. The findings indicate that TFPI-2 reduces the invasive and migratory capabilities of tumor cells by interacting with PSAP, offering a novel viewpoint and potential target for fibrosarcoma treatment [26] (Figure 6).

In summary, PSAP emerges as a significant molecule across diverse cancer types, demonstrating dual potential as a diagnostic/prognostic biomarker and a therapeutic target. Its expression is frequently dysregulated in malignancies, correlating with aggressive disease features and poor clinical outcomes. PSAP serves as a confirmed biomarker in breast cancer with elevated expression linked to poor prognosis, in GC with tissue overexpression associated with advanced stage and adverse outcomes, and in CRC as a CMS4/CRIS-B subtype marker; potential biomarkers to be confirmed, which include its role as a serum staging marker in PCa, an early diagnostic marker in GBC, and a co-diagnostic marker with QSOX1 in mesothelioma. For ovarian cancer, gliomas, glioblastoma, hepatocellular carcinoma, and fibrosarcoma, PSAP participates in key pathways but requires further validation as a biomarker. Functionally, PSAP drives oncogenesis through multiple mechanisms: it enhances ERα signaling in breast cancer, promotes AR activity and cell survival in PCa, drives glioma stem cell growth via TLR4/NF-κB signaling in gliomas, facilitates invasion and EMT in glioblastoma via TGF-β1/Smad, supports proliferation in hepatocellular carcinoma via the circVAPA/miR-377-3p axis, and contributes to an immunosuppressive tumor microenvironment in PDAC by inhibiting CD8^+^ T-cell infiltration. Paradoxically, in breast cancer, PSAP secreted by mesenchymal stem cells may suppress metastasis while promoting primary tumor cell survival. Similarly, in ovarian cancer, PSAP-derived peptides inhibit tumor growth and metastasis via TSP-1/CD36 signaling. In fibrosarcoma, PSAP-TFPI-2 interaction suppresses tumor progression. Therapeutically, targeting PSAP holds promise: a cyclic PSAP-derived peptide reactivates anti-tumor TSP-1/CD36 signaling to inhibit ovarian cancer growth and metastasis in vivo. TLR4 inhibition blocks PSAP-driven proliferation in gliomas, and inhibiting the PSAP/TGF-β1/Smad axis suppresses invasive growth in glioblastoma, and modulating PSAP expression enhances CD8^+^ T-cell responses against pancreatic cancer. PSAP’s interaction with TFPI-2 also presents a vulnerability in fibrosarcoma. Collectively, the evidence positions PSAP as a versatile pan-cancer biomarker with tissue-specific role in oncogenesis and therapeutic potentials (Table 1). Critically, its context-dependent duality (promoting or suppressing tumor progression) likely stems from distinct microenvironmental factors and signaling pathway activation that differentially regulate PSAP function–a complex interplay which remains incompletely understood and warrants deeper mechanistic investigation. This positions PSAP as a compelling candidate for further development in cancer diagnostics and targeted therapies.

## 4. Discussion and Conclusions

In this review, we provide insights into the roles of prosaposin (PSAP) and its derived saposins in a variety of biological processes, as well as their pathological roles in different diseases. PSAP is a multifunctional protein that plays a variety of intra- and extracellular roles, including the activation of enzymes in lysosomes, the regulation of neuronal cell survival and growth, the participation in regulation of immune reactions and tumorigenesis.

The role of PSAP is particularly important in the nervous system. PSAP, as an important neurotrophic factor, plays a key role in neuronal cell survival, differentiation, neuronal synapse growth, and synapse formation, and regulates the transport and distribution of ganglioside GM1 by working together with Sap A–D. PSAP and its derivative Sap C exert neuroprotective effects by regulating the level of α-Syn to attenuate the pathological features of neurodegenerative diseases, while activating GPR37L1 and GPR37 receptors to protect astrocytes from oxidative stress damage and enhance their protective effects on damaged neurons. In metabolic regulation, PSAP is upregulated in the early stage of adipocyte differentiation and regulates systemic energy metabolism and immune homeostasis by binding to CD1d to present lipid antigens to iNKT cells. At the same time, PSAP acts as a CoQ10-binding protein to maintain intracellular CoQ10 levels, which was essential for cellular energy metabolism and antioxidant capacity. In reproductive function, the interaction of PSAP and Rhox5 may have a regulatory role in the development of prostasomal reproductive organs, spermatogenesis, and fertilization ability through MAPK and PI3K/Akt signaling pathways. In immune regulation, PSAP is upregulated in M2 macrophages, where it mediates immunomodulation, anti-inflammatory responses, antioxidant defense, and tissue repair. Furthermore, PSAP synergistically activates sphingolipid metabolism (e.g., S-1-P production) through coordinated ERK, SK, and PI3K/Akt signaling pathways, driving cell proliferation and anti-apoptosis to establish its central role in cellular survival and metabolic homeostasis. In addition, PSAP may facilitate lysosomal antigen presentation in the tumor microenvironment to enhance anti-tumor immunity.

The pathophysiological roles of PSAP and its derived saposins are not executed in isolation but are deeply embedded within intricate cellular signaling networks, engaging in dynamic crosstalk with key molecular players and pathways. This context-dependent interplay significantly dictates the functional complexity and often contrasting outcomes of PSAP in specific diseases. Within neurodegenerative disorders, this crosstalk is particularly evident. In PD, PSAP/Sap C not only regulates GCase activity but also exhibits direct or indirect antagonism with α-Syn—Sap C competitively binds GCase, preventing α-Syn-mediated inhibition of GCase activity, while PSAP overexpression reduces α-Syn levels. Concurrently, PSAP cooperatively interacts with PGRN within lysosomes, jointly influencing GCase trafficking and activation, forming a critical regulatory axis in PD pathology. In AD, the early interaction between PSAP and PGRN promotes PGRN oligomerization and sequestration within Aβ plaques, diminishing its neuroprotective and anti-inflammatory functions. Conversely, in late AD stages dominated by tau pathology, reduced PSAP expression and attenuated PSAP-PGRN interaction are strongly linked to accelerated NFT formation. For GD, the core interaction centers on PSAP/Sap C and GCase: Sap C, as an essential cofactor, maintains GCase activity by stabilizing its structure and facilitating its binding to anionic phospholipid-rich lysosomal membranes. Mutations in the *PSAP* gene disrupting Sap C production catastrophically break this interaction, leading to substrate accumulation and disease manifestations. In AS, the pro-inflammatory role of PSAP is tightly coupled to the mTOR/S6K1 signaling pathway; its expression is induced by this pathway and mediates macrophage inflammatory responses and glycolysis/oxidative phosphorylation. Extracellularly, PSAP (via Effero-EVs) binds the GPR37 receptor on macrophages, activating ERK/c-Fos signaling to upregulate Tim4 expression and enhance efferocytosis, illustrating PSAP’s bidirectional role in modulating inflammation-resolution balance. Within the realm of cancer, PSAP’s interaction networks are highly tumor-type specific. In breast cancer, PSAP drives estrogen-dependent growth by upregulating ERα expression and activity via the MAPK pathway, and its secretion is influenced by crosstalk with the tumor microenvironment (e.g., ECM stiffness signals from CAFs). In PCa, PSAP/Sap C engages in a robust positive feedback loop with the AR signaling axis—androgens induce PSAP transcription, while PSAP/Sap C enhances AR activity and downstream gene expression (e.g., *PSA*) in a ligand-independent manner, synergizing with integrin, uPA/uPAR, MAPK/JNK, and PI3K/Akt pathways to promote tumor progression. In ovarian cancer, tumor microenvironment suppression of PSAP leads to reduced secretion of its downstream TSP-1, whereas therapeutic PSAP cyclic peptide reactivates the TSP-1-CD36 anti-tumor axis by activating bone marrow-derived monocytes. In GBM, PSAP promotes invasion and EMT through TLR4/NF-κB and TGF-β1/Smad pathways. In PDAC, secreted PSAP critically interacts with the immune microenvironment by suppressing CD8^+^ T-cell infiltration. These diverse examples underscore the extreme context-dependency of PSAP function. Delving deeper into the intricate interaction networks of PSAP with specific partner molecules (e.g., α-Syn, PGRN, GCase, ERα, AR, TSP-1, TLR4, TGF-β, GPR37, mTOR) and core pathways (e.g., MAPK, PI3K/Akt, ERK, NF-κB, Smad, lysosomal-autophagy pathways) across different disease contexts is paramount not only for a comprehensive understanding of its multifaceted roles in pathogenesis but also for identifying novel, interaction-specific targets for the development of more precise therapeutic interventions.

The clinical translational potential of PSAP is substantial and multifaceted, rooted in its dual roles as a critical biomarker and an innovative therapeutic target. Its secretory nature facilitates detection in bodily fluids, underpinning its value in non-invasive diagnostics. For instance, serum PSAP is significantly elevated in MPM, and combining it with QSOX1 enhances diagnostic accuracy. Strikingly, 83% of GBC tissues exhibit strong PSAP positivity, suggesting its utility as a histopathological marker. Crucially, PSAP expression levels possess robust prognostic power: elevated serum PSAP distinguishes mCRPCa from localized disease and correlates with advanced tumor stage and reduced overall survival in GC, while high expression associates with poor-prognosis molecular subtypes and worse survival outcomes in CRC. As a therapeutic target, diverse intervention strategies show promise. Direct targeting of PSAP or its signaling axes is effective: a cyclic PSAP-derived peptide reactivates the anti-tumor TSP-1/CD36 axis by activating monocytes, inhibiting ovarian cancer growth and metastasis in PDX models; inhibiting the PSAP-TLR4/NF-κB pathway blocks glioma progression. Modulating PSAP expression (e.g., downregulation in PDAC to boost CD8^+^ T-cell infiltration) or leveraging its physiological functions (e.g., enhancing PSAP/Sap C levels to sustain GCase activity in PD) represent significant therapeutic avenues.

However, translating this promise faces a core bottleneck: the inherent risk of complex off-target effects stemming from PSAP’s fundamental multifunctionality. PSAP and its derived saposins are central to essential physiological processes including neuronal survival (e.g., via GPR37 receptor), lysosomal homeostasis, immunomodulation, and energy balance. Inhibiting pathogenic functions (e.g., the PSAP/AR axis in PCa) risks inadvertently disrupting these vital roles, such as neuroprotection or lysosomal integrity. Compounding this challenge are the contradictory roles of specific molecular isoforms/fragments. Sap C drives tumor progression in PCa but is indispensable for GCase function in GD and PD, making selective targeting perilous. Furthermore, PSAP’s extensive interaction network (e.g., its KD2 domain binds TFPI-2 in fibrosarcoma while its C-terminus interacts with Sortilin for lysosomal trafficking) creates vulnerability where blocking one interaction may unintentionally disrupt another critical function. Overcoming these obstacles necessitates the development of highly precise intervention strategies. Cutting-edge technologies offer solutions: Proteolysis-Targeting Chimeras (PROTACs) [190] act as “molecular scalpels,” selectively degrading disease-specific PSAP complexes (e.g., oncogenic PSAP-AR) while sparing physiological forms (e.g., neuroprotective PSAP-GPR37). Developing isoform-specific antibodies that distinguish pathogenic conformations (e.g., cancer-promoting Sap C) from protective full-length PSAP, and advancing intelligent delivery systems (e.g., pH/enzyme-responsive nanocarriers) that restrict therapeutic activity to pathological sites, are crucial for minimizing off-target effects and achieving functional selectivity. Strategic differentiation of PSAP’s dual roles is paramount for successful clinical translation.

In conclusion, PSAP and its derived saposins play complex and diverse roles in a variety of biological processes. Their important roles in the nervous system, immune system, cardiovascular diseases, and cancers provide potential targets for future therapeutic strategies. However, the multiple roles of PSAPs in different diseases also suggest the need to carefully consider their specific mechanisms of action in specific disease contexts when developing PSAP-based therapeutic strategies. Future studies are needed to further explore the role of PSAP in various diseases in order to better understand its functions in different pathological processes and provide new strategies for disease treatment.

## Figures and Tables

**Table 1:** The critical role of PSAP in cancer.

Cancer Type	Key Pathway/ Mechanism	Biomarker	Therapeutic Potential	Role in Tumor	Reference
Breast Cancer	ERα/MAPK signaling	Prognostic marker	Inhibition of miR-23b/27b/24/PSAP axis	Pro-tumor/Anti-tumor	[17,157,158]
Ovarian Cancer	TSP-1/CD36 apoptosis pathway	Unknown	Cyclic PSAP peptide	Anti-tumor	[160]
Prostate Cancer	AR signaling axis	Serum staging marker (TBC)	Blocking PSAP/AR axis	Pro-tumor	[18,167]
Gliomas	TLR4/NF-κB signaling	Unknown	Inhibition of the PSAP/TLR4/NF-κB axis via TLR4 inhibitors	Pro-tumor	[19]
Glioblastoma	TGF-β1/Smad- induced EMT	Unknown	Inhibiting PSAP/TGF-β/Smad axis	Pro-tumor	[175]
Gastric Cancer	PDCD1/TGFB1/CSF1R inhibition	Prognostic marker	Inhibiting PSAP/Immune checkpoint axis	Pro-tumor	[20]
Colorectal Cancer	dMMR/CIMP+/BRAF mutations	CMS4/CRIS-B subtype marker	Unknown	Pro-tumor	[21]
Gallbladder Cancer	Unknown	Early diagnostic marker (TBC)	Unknown	Pro-tumor	[22]
Hepatocellular carcinoma	circVAPA/miR-377-3p axis	Unknown	Inhibition of PSAP by miR-377-3p	Pro-tumor	[23]
Pancreatic ductal adenocarcinoma	CD8^+^ T-cell inhibition	Immune microenvironment marker (TBC)	Blocking PASP-induced immunosuppression	Pro-tumor	[24]
Malignant pleural mesothelioma	Oxidative stress defense	Co-diagnostic marker with QSOX1 (TBC)	Unknown	Pro-tumor	[25]
Fibrosarcoma	TFPI-2/PSAP/MMP2 pathway	Unknown	Inhibition of PSAP/MMP2 axis by TFPI-2	Pro-tumor	[26]

TBC: to be confirmed.

## Data Availability

No new data was generated during this study.

## Abbreviations

Aβ	Amyloid beta
AUC	Analytical ultracentrifugation
AD	Alzheimer’s disease
ADPD	Autosomal dominant Parkinson’s disease
AI	Androgen-independent
AR	Androgen receptor
ARs	Activity of hormone receptors
ASMase	Acid sphingomyelinase
α-Syn	α-synuclein
BMP	bis (monoacylglycero) phosphate
CAFs	Cancer-associated fibroblasts
CTSB	Cathepsin B
CTSD	Cathepsin D
CMS4	Consensus molecular subtype 4
CoQ	Coenzyme Q (ubiquinone)
CRC	Colorectal cancer
CRPC	Castration-resistant prostate cancer
CRIS-B	Colorectal cancer intrinsic subtype B
CSF	Cerebrospinal fluid
CSPC	Castration-sensitive prostate cancer
DA	Dopamine
DNs	Dystrophic neurites
EC	Endothelial cell
ECB	Encapsulated cell biodelivery
EMT	Epithelial–mesenchymal transition
EOPD	Early-onset Parkinson’s disease
ER	Estrogen receptor
ER α	Estrogen receptor α
ERKs	Extracellular signal-regulated kinases
ERK	Extracellular signal-regulated kinase
EVs	Extracellular vesicles
FPD	Familial Parkinson’s disease
FTD	Frontotemporal dementia
GalEAG	Galactosylalkylacylglycerol
Gb3	Globotriaosylceramide
GD	Gaucher disease
GBC	Gallbladder cancer
GCase	Glucocerebrosidase
GC	Gastric cancer
Gln	Glucosamine
GSL	Glycosphingolipids
GM1	Ganglioside GM1
GPR37	G protein-coupled receptor 37
GPR37L1	G protein-coupled receptor 37L1
HCC	Hepatocellular carcinoma
HPLC	High-performance liquid chromatography
HRE	Hormone-responsive element
KO	Knockout
KD2	Second Kunitz-type domain
LacCer	Lactoceramide
LAMP1	Lysosome-associated membrane protein 1
LIMP-2	Lysosomal integral membrane protein 2
LPC	Localized prostate cancer
LRP	Lipoprotein receptor-associated protein
LC-MS/MS	Liquid chromatography-tandem mass spectrometry
LSDs	Lysosomal storage disease
M6P	Mannose-6-phosphate
MASLD	Metabolic dysfunction-associated steatotic liver disease
MCI	Mild cognitive impairment
MEK	Mitogen-activated extracellular signal-regulated kinase
mCRPCa	Metastatic castrate-resistant prostate cancer
MPC	Metastatic prostate cancer
MPM	Malignant pleural mesothelioma
MMP-2	Matrix metalloproteinase-2
NFTs	Neurofibrillary tangles
OXPHOS	Oxidative phosphorylation
ox-LDL	Oxidized low-density lipoprotein
PCa	Prostate cancer
PD	Parkinson’s disease
PDAC	Pancreatic ductal adenocarcinoma
PGRN	Progranulin
PI3K	Phosphatidylinositol 3-kinase
POPC	Palmitoyloleoylphosphatidylcholine
PrSt	Prostate stromal cells
proCath-D	Procathepsin D
PSAP	Prosaposin
QSOX1	Quiescin Q6 sulfhydryl oxidase 1
RAP	Receptor-associated protein
ROS	Reactive oxygen species
S-1-P	Sphingosine-1-phosphate
Sap A	Saposin A
Sap B	Saposin B
Sap C	Saposin C
Sap D	Saposin D
SAPK/JNK	Stress-activated protein kinase/c-jun n-terminal kinase
SG	Spiral ganglion
SK	Sphingosine kinase
SGP-1	Sulfated glycoprotein-1
SPD	Sporadic Parkinson’s disease
STAD	Stomach adenocarcinoma
TCGA	The Cancer Genome Atlas
TFPI-2	Tissue factor pathway inhibitor-2
TGF-β	Transforming growth factor-β
TGF-β1/Smad	Transforming growth factor-β1/Smad
TISIDB	Tumor Immune System Interaction Database
TMT	Tandem mass spectrometry tagging
TNFα	Tumor necrosis factor alpha
TSP-1	Anti-tumor protein thrombospondin-1
uPA	Urokinase-type fibrinogen activator
uPAR	Urokinase-type fibrinogen activator receptor
UPR	Unfolded protein response
6-OHDA	6-hydroxydopamine
